# Patients with Chronic Spinal Cord Injury and a Long Period of Evolution Exhibit an Altered Cytokine Production by CD4 and CD8 T Cell Populations

**DOI:** 10.3390/ijms24087048

**Published:** 2023-04-11

**Authors:** Sergio Haro Girón, Ana M. Gómez-Lahoz, Jorge Monserrat Sanz, Oscar Fraile-Martínez, Diego J. Jiménez, Cielo Garcia-Montero, Diego de Leon-Oliva, Miguel A. Ortega, Mar Atienza-Perez, David Diaz, Elisa Lopez-Dolado, Melchor Álvarez-Mon

**Affiliations:** 1 Department of Medicine and Medical Specialities, Faculty of Medicine and Health Sciences, University of Alcalá, 28801 Alcalá de Henares, Spain; 2 Ramón y Cajal Institute of Sanitary Research (IRYCIS), 28034 Madrid, Spain; 3 Service of Rehabilitation, National Hospital for Paraplegic Patients, Carr. de la Peraleda, S/N, 45004 Toledo, Spain; 4 Service of Internal Medicine and Immune System Diseases-Rheumatology, University Hospital Príncipe de Asturias (CIBEREHD), 28806 Alcalá de Henares, Spain

**Keywords:** chronic spinal cord injury (SCI), T lymphocytes, interleukin 10 (IL-10), interleukin 9

## Abstract

Spinal cord injury (SCI) is a disabling neurological condition coursing with serious multisystem affections and morbidities. Changes in immune cell compartments have been consistently reported in previous works, representing a critical point of study for understanding the pathophysiology and progression of SCI from acute to chronic stages. Some relevant variations in circulating T cells have been noticed in patients with chronic SCI, although the number, distribution, and function of these populations remain to be fully elucidated. Likewise, the characterization of specific T cell subpopulations and their related cytokine production can aid in understanding the immunopathological role of T cells in SCI progression. In this sense, the objective of the present study was to analyze and quantify the total number of different cytokine-producers T cells in the serum of patients with chronic SCI (*n* = 105) in comparison to healthy controls (*n* = 38) by polychromatic flow cytometry. Having this goal, we studied CD4 and CD8 lymphocytes as well as naïve, effector, and effector/central memory subpopulations. SCI patients were classified according to the duration of the lesion in chronic SCI with a short period of evolution (SCI-SP) (comprised between 1 and 5 years since initial injury), early chronic phase (SCI-ECP) (between 5 and 15 years since initial injury) and late-chronic phase (SCI-LCP) (>15 years since initial injury). Our results show that patients with chronic SCI exhibited an altered immune profile of cytokine-producer T cells, including CD4/CD8 naïve, effector, and memory subpopulations in comparison to HC. In particular, IL-10 and IL-9 production seems to be importantly altered, especially in patients with SCI-LCP, whereas changes in IL-17, TNF-α, and IFN-γ T cell populations have also been reported in this and other chronic SCI groups. In conclusion, our study demonstrates an altered profile of cytokine-producer T cells in patients with chronic SCI, with marked changes throughout the course of the disease. In more detail, we have observed significant variations in cytokine production by circulating naive, effector, and effector/central memory CD4 and CD8 T cells. Future studies should be directed to explore the possible clinical consequences of these changes or develop additional translational approaches in these groups of patients.

## 1. Introduction

A spinal cord injury (SCI) is severe neurological damage in the spinal cord that frequently courses various comorbidities and permanent disability [1]. Generally, this condition occurs because of a sudden, traumatic event that fractures or dislocates vertebrae (mainly due to automobile crashes and falls), although certain medical or surgical complications can also cause SCI [2]. The pathophysiological basis of SCI remains to be fully unraveled. After trauma, the initial mechanical injury directly disrupts neuronal axons, blood vessels, and cell membranes (primary injury). This initial stage is followed by a secondary injury, implicating vascular dysfunction, ischemia, edema, excitotoxicity, electrolyte shifts, oxidative stress, inflammation, and delayed apoptotic cell death [3]. This secondary injury is subdivided into the acute phase (during the first 48 h), sub-acute phase (until 2 weeks), and chronic phase (which extends from days to years). The pathophysiological events occurring in each phase are different, the chronic phase being the largest period for SCI patients [4]. Importantly, individuals with chronic SCI tend to accumulate not only spinal but also systemic alterations, which can be related to the onset of many comorbidities, representing an important medical challenge [5]. To this fact, the high socioeconomic burden of SCI must be added, with prominent direct and indirect costs related [6,7]. Thus, a greater understanding of the pathophysiological signatures involved in chronic SCI is required to describe and mitigate the consequences of this systemic malady.

The immune system represents a pivotal point of study with multiple functions in chronic SCI. The inflammatory process is critically involved in the downstream reactions following direct SCI injury. It is now broadly accepted that inflammation has a dual role in SCI, exerting beneficial actions (i.e., improving tissue plasticity and remodeling) but also detrimental ones, participating in the secondary injury cascade [8]. In patients with chronic SCI, profound immune dysregulation can be observed, as SCI impairs the neural and humoral control of immune cells [9]. Eventually, immune dysfunction can lead to the development of systemic inflammatory response syndrome (SIRS), compensatory anti-inflammatory response syndrome (CARS), and SCI-immune depression syndrome (SCI-IDS) [10]. Likewise, other immune-related phenomena such as autoimmunity, low-grade chronic inflammation, or immunosenescence can also be observed in SCI patients [11,12,13]. Therefore, SCI-related immune dysfunction is involved in the many comorbidities that occur in these subjects [14]. Indeed, the risk of various medical problems and readmission rates increase by approximately 37% per year, especially due to urinary tract infections (UTIs) [15]. In this sense, deepening the study of the immune system is essential to improve the clinical management of patients with chronic SCI, aiding in understanding this complex entity.

The study of specific immune subsets obtained from the peripheral blood of patients with chronic SCI has received increasing attention in recent years. Along these lines, previous works have identified altered levels of multiple serum cytokines and immune populations in patients with SCI [16,17]. For instance, we showed that enhanced markers of intestinal barrier dysfunction and inflammatory markers such as tumor necrosis factor-alpha (TNF-α) and interleukin 6 (IL-6) can be observed in patients with chronic SCI, which are associated with impaired function of circulating monocyte [18]. Furthermore, the intensity and characteristics of the immune dysfunction seem to vary throughout the course of the disease [19]. T cells are specific subsets of lymphocytes characterized by being positive in the CD3 marker, playing a central role in the adaptative immune response under homeostatic and disease conditions [20]. T cells can be mainly classified into two main subpopulations recognized as CD4 and CD8 lymphocytes. Initially, these populations are naïve. Then, they become effectors when they are stimulated after antigen recognition, and some of them become memory T cells with rapid recognition of their specific antigens [21]. These cells can produce a broad spectrum of proinflammatory and anti-inflammatory cytokines, aiding in orchestrating the immune responses [22]. Previous studies have shown marked alterations in different subpopulations of circulating CD4 and CD8 in patients with SCI, including naïve, effector, and memory cells [23,24,25,26]. However, despite the fact that marked dysregulation of some of the cytokines has been demonstrated in the serum of patients with SCI [27], to our knowledge, there are no studies evaluating neither cytokine production by specific T cell subpopulations nor their ability to produce cytokines after being stimulated.

Thus, the aim of the present work is to study cytokine production by specific T cell subsets (including total T CD4/CD8, naïve, and memory cells) in basal conditions and after stimulation in patients with chronic SCI and compare them with healthy controls. Moreover, we will classify chronic SCI subjects according to the time since initial injury in three groups: chronic SCI with a short period of evolution (SCI-SP), if the time since initial injury was comprised between 1 and 5 years; chronic SCI at early chronic phase (SCI-ECP) if the time of evolution was between 5 and 15 years; and chronic SCI at late-chronic phase (SCI-LCP) if the time of evolution was more than 15 years.

## 2. Results

### 2.1. Patients Demographics

We studied 101 patients (mean age 35.23 ± 12.84% years and years 70.10% men) with chronic SCI and 40 healthy controls (HC; 32.74 ± 8.92 years 63.70% men). The mean time of SCI onset was 12.99 ± 9.16.

The neurological level of spinal damage was located within C1–C4, C5–C8, T1–T6, T7–T12, and the lumbosacral metamers in 23.8%, 20%, 26.27%, 20.95%, and 8.57% of patients, respectively. In other words, more than 70.40% of our patients had an SCI above T6. With respect to the ASIA, 46.67% of the patients were AIS A, 16.19% of the patients were AIS B, 16.19% of the patients were AIS C, and 20.95% of the patients were AIS D, indicating that although 79.04% of the patients exhibit incomplete lesions, just 62.85% reported incomplete motor injuries, with different extent of intralesional motor preservation and theoretically better mobility profiles. Overall, the demographic data of our chronic SCI patients and HC are collected in Table 1.

### 2.2. Patients with Chronic Spinal Cord Injury and a Long Period of Evolution Exhibit an Altered Cytokine Production by Total, Naïve, Effector, and Central/Effector Memory CD4+ Cells

We investigated the pattern of IFN-γ, IL-10, IL-17, IL-9, TNF-α, and IL-2 production by CD4 lymphocytes and their different subpopulations from 101 chronic SCI patients classified according to the period of evolution (SCI-SP, SCI-ECP, and LCP) and compared it with 40 sex- and age-matched HCs. Herein, we will subdivide the main results into different subsections, comparing the outcomes obtained at basal conditions (medium) and after PMA stimulation. Representative FACS plots indicating T cell subsets and the intracellular cytokine staining are shown in Appendix A, Appendix B, Appendix C and Appendix D.

#### 2.2.1. Cytokine Expression in CD4 Lymphocytes

**Figure 1:**
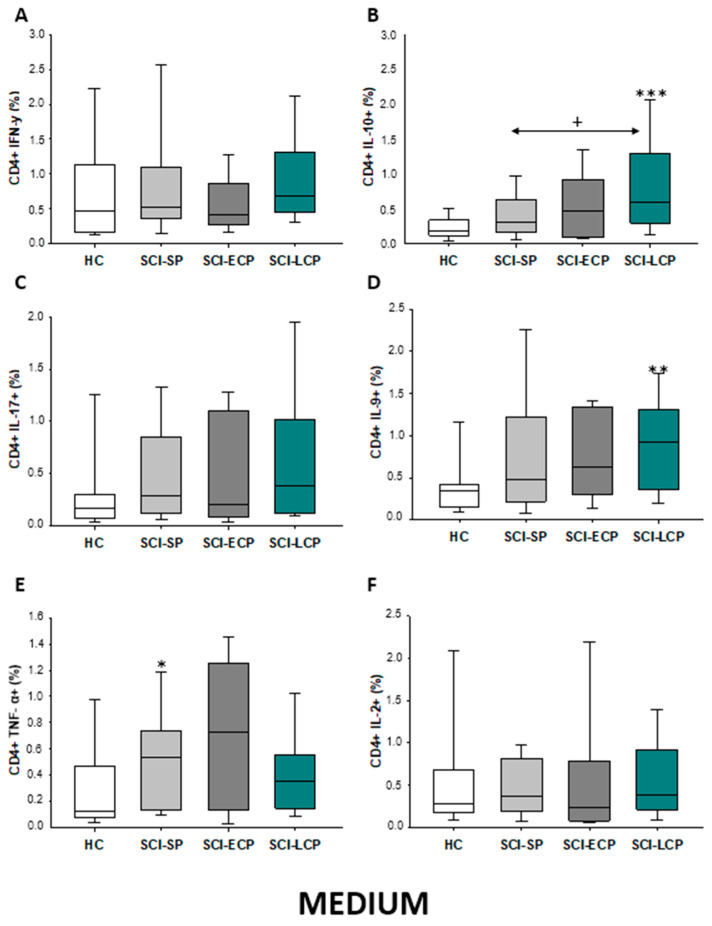
(**A**–**F**) Spontaneous cytokine expression by CD4 lymphocytes from SCI patients and HC. Percentage of CD4 cells producing IFN-γ, IL-10, IL-17, IL-9, TNF-α, and IL-2 in healthy controls (HC); chronic SCI patients with short periods of evolution (<5 years) (SCI-SP); chronic SCI patients in early chronic phase (5 to 15 years) (SCI-ECP); and chronic SCI patients in late-chronic phase (>15 years) (SCI-LCP). We use ‘*’ to distinguish between chronic SCI patients and HC, whereas ‘+’ is used to compare chronic SCI patients. *p* < 0.05 (*/+), *p* < 0.01 (**), and *p <* 0.001 (***).

Firstly, we evaluated the spontaneous expression of IFN-γ, IL-10, IL-17, IL-9, TNF-α, and IL-2 by CD4 lymphocytes from SCI patients and HC. We observed that patients with SCI-LCP exhibit a significant increase in the spontaneous expression of IL-10 by CD4 cells in comparison to HC (SCI-LCP = 0.56 [0.3–1.29]; HC = 0.19 [0.125–0.34], *** *p* < 0.001); and SCI-SP (SCI-SP = 0.305 [0.165–0.565], * *p* = 0–0.46). (Figure 1). Similarly, our results report a marked increase in IL-9 production in patients with SCI-LCP when compared to HC (SCI-LCP = 0.93 [0.36–1.31]; HC = 0.345 [0.15–0.42], ** *p* = 0.008) as well as in TNF-α production by SCI-SP in comparison to HC (SCI-SP = 0.535 [0.140–0.735]; HC = 0.130 [0.078–0.460], * *p* = 0.035).

**Figure 2:**
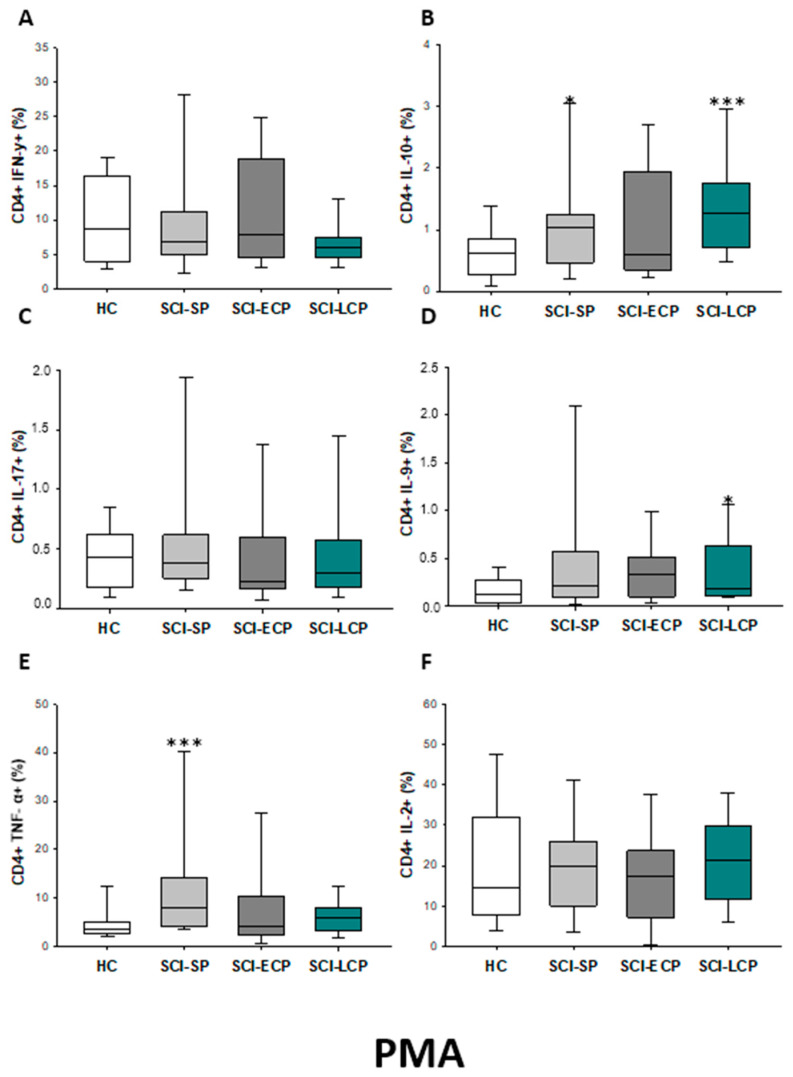
(**A**–**F**) Cytokine expression by PMA stimulated CD4 lymphocytes from SCI patients and HC. Percentage of CD4 cells producing IFN-γ, IL-10, IL-17, IL-9, TNF-α, and IL-2 in healthy controls (HC) after PMA stimulation; chronic SCI patients with short periods of evolution (<5 years) (SCI-SP); chronic SCI patients in early chronic phase (5 to 15 years) (SCI-ECP); and chronic SCI patients in late-chronic phase (>15 years) (SCI-LCP). We use ‘*’ to distinguish between chronic SCI patients and HC. *p* < 0.05 (*) and *p <* 0.001 (***).

We also investigated the IFN-γ, IL-10, IL-17, IL-9, TNF-α, and IL-2 expression by CD4 lymphocytes after PMA stimulation. (Figure 2). We observed that there was a significant increase in the percentage of CD4 IL-10 producers in SCI-LCP when compared to HC (SCI-LCP = 0.127 [0.7–1.76]; HC = 0.625 [0.27–0.83], *** *p* = 0.001) and similar variations could be reported in term of IL-9 production (SCI-LCP = 0.19 [0.11–0.63]; HC = 0.13 [0.045–0.265], * *p* = 0.043). On the other hand, CD4 cells in SCI-SP also produce higher levels of IL-10 than the HC (SCI-SP = 1.045 [0.51–1.225], * *p* = 0.026) and a more marked TNF-α production (SCI-SP = 7.98 [4.42–14.10]; HC = 3.68 [2.78–5.07], ** *p* = 0.004).

#### 2.2.2. Cytokine Expression in CD4 Naïve Lymphocytes

**Figure 3:**
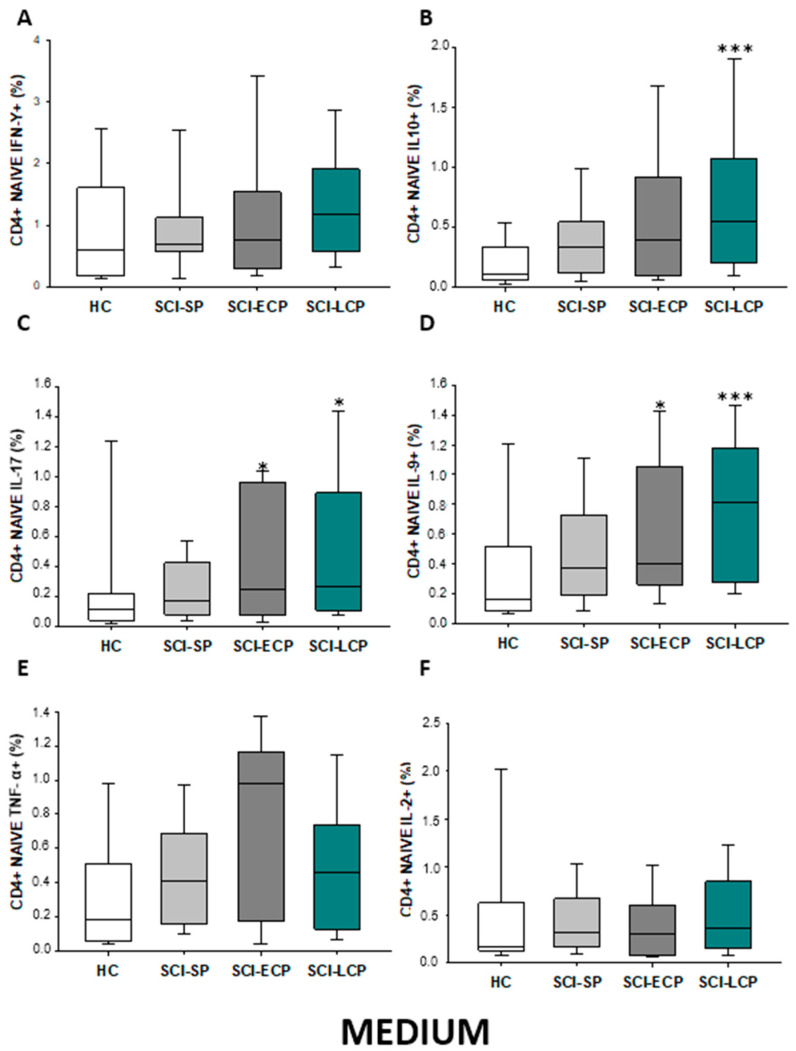
(**A**–**F**) Percentage of CD4 naïve cells producing IFN-γ, IL-10, IL-17, IL-9, TNF-α, and IL-2 in healthy controls (HC); chronic SCI patients with short periods of evolution (<5 years) (SCI-SP); chronic SCI patients in early chronic phase (5 to 15 years) (SCI-ECP); and chronic SCI patients in late-chronic phase (>15 years) (SCI-LCP). We use ‘*’ to distinguish between chronic SCI patients and HC. *p* < 0.05 (*) and *p <* 0.001 (***).

Regarding CD4 naïve populations (Figure 3A–F), we observed in SCI-LCP patients a significant increase in the production of IL-10 when compared to HC (SCI-LCP= 0.9 [0.47–1.59]; HC = 0.462 [0.195–0.785], *** *p* ≤ 0.001), IL-17 (SCI-LCP = 0.17 [0.083–0.45]; HC = 0.0825 [0.0455–0.26], * *p* = 0.022). A concomitant increase was reported in patients with SCI-LCP when assessing IL-9 production (SCI-LCP = 0.17 [0.088–0.44]; HC = 0.0885 [0.0305–0.21], *** *p* < 0.001). Likewise, a significant increment was equally defined in SCI-ECP patients in terms of IL-9 production (SCI-ECP = 0.15 [0.0955–0.315], *p* = 0.047).

**Figure 4:**
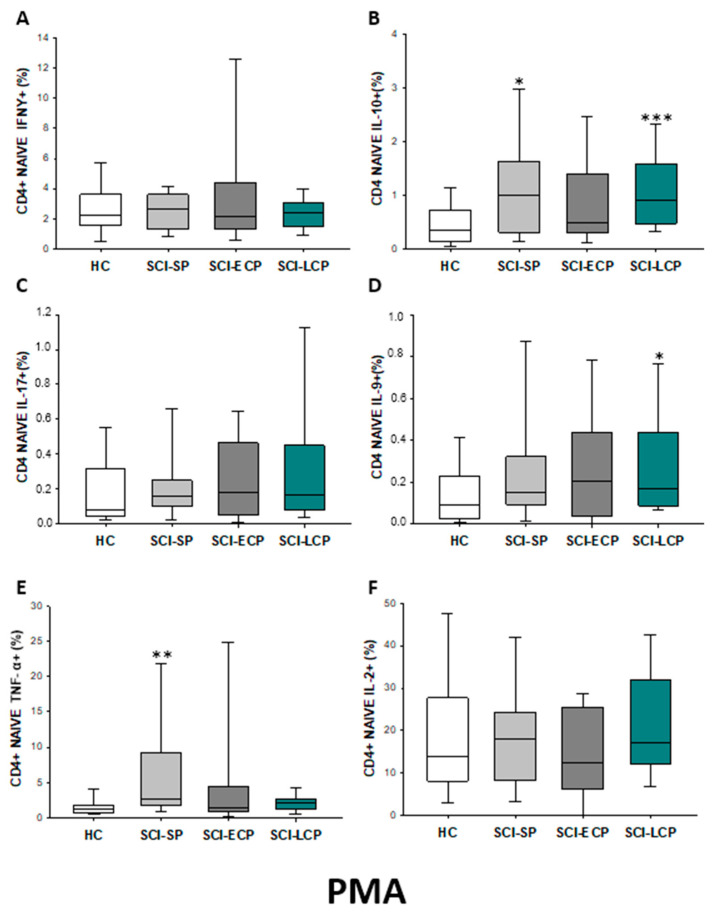
(**A**–**F**) Percentage of CD4 naïve cells producing IFN-γ, IL-10, IL-17, IL-9, TNF-α, and IL-2 in healthy controls (HC) after PMA stimulation; chronic SCI patients with short periods of evolution (<5 years) (SCI-SP); chronic SCI patients in early chronic phase (5 to 15 years) (SCI-ECP); and chronic SCI patients in late-chronic phase (>15 years) (SCI-LCP). We use ‘*’ to distinguish between chronic SCI patients and HC. *p* < 0.05 (*), *p* < 0.01 (**), and *p <* 0.001 (***).

After PMA stimulation (Figure 4A–F), a significant increase in IL-10 production by CD4 naïve cells could be observed in SCI-LCP (SCI-LCP = 0.17 [0.083–0.45]; HC = 0.0825 [0.0455–0.26], ** *p* = 0.002) as it occurs with IL-9 production (SCI-LCP = 0.17 [0.088–0.44]; HC = 0.0885 [0.0305–0.21], * *p* = 0.026). Moreover, SCI-SP patients also exhibited higher production of IL-10 (SCI-SP = 0.16 [0.1–0.25], * *p* = 0.019) and TNF-α than HC (SCI-SP = 2.67 [1.84–7.61]; HC = 1.21 [0.74–1.68], ** *p* = 0.003.

#### 2.2.3. Cytokine Expression in CD4 Effector Lymphocytes

**Figure 5:**
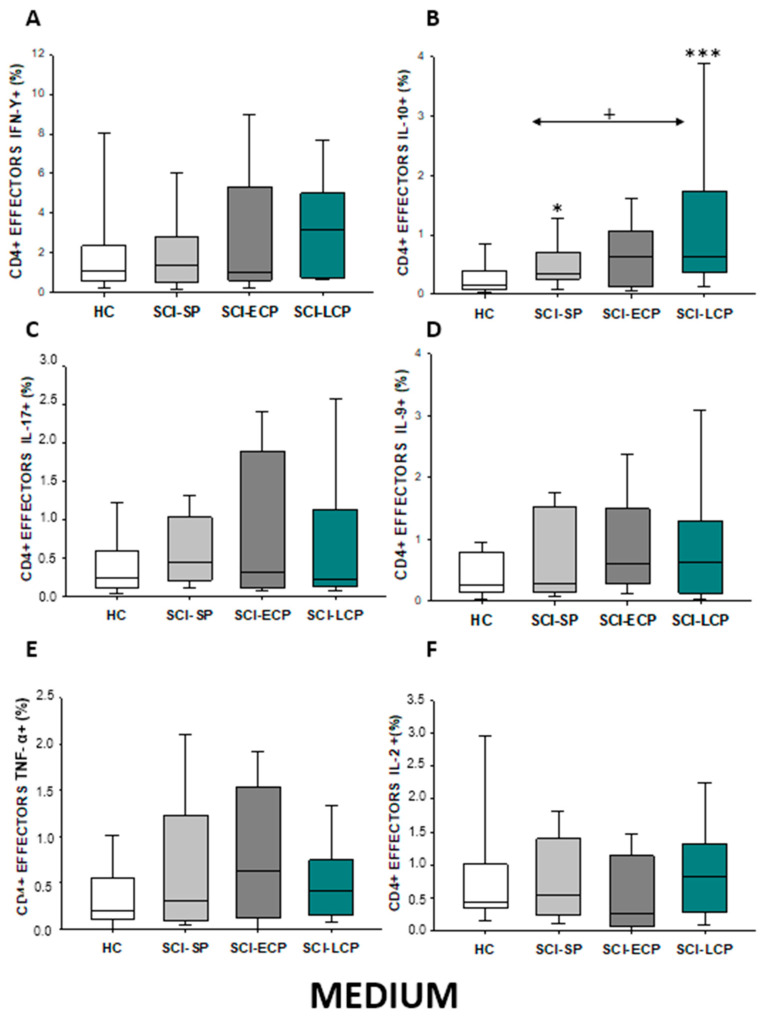
(**A**–**F**) Percentage of CD4 effector cells producing IFN-γ, IL-10, IL-17, IL-9, TNF-α, and IL-2 in healthy controls (HC); chronic SCI patients with short periods of evolution (<5 years) (SCI-SP); chronic SCI patients in early chronic phase (5 to 15 years) (SCI-ECP); and chronic SCI patients in late-chronic phase (>15 years) (SCI-LCP). We use ‘*’ to distinguish between chronic SCI patients and HC, whereas ‘+’ is used to compare chronic SCI patients. *p* < 0.05 (*/+) and *p <* 0.001 (***).

In the event of effector CD4 lymphocytes (Figure 5A–F), our results show that there was a significant increase in IL-10 production observed in SCI-LCP patients when compared to healthy subjects (SCI-LCP = 0.64 [0.37–1.74]; HC = 0.165 [0.087–0.37], *** *p* = 0.01). Moreover, there is a similar increase in the production of this cytokine from patients with SCI-SP and healthy controls (SCI-SP = 0.35 [0.26–0.71], * *p* = 0.05). However, this increase was significantly higher in patients with SCI-LCP when compared to SCI-SP (+ *p* = 0.041).

**Figure 6:**
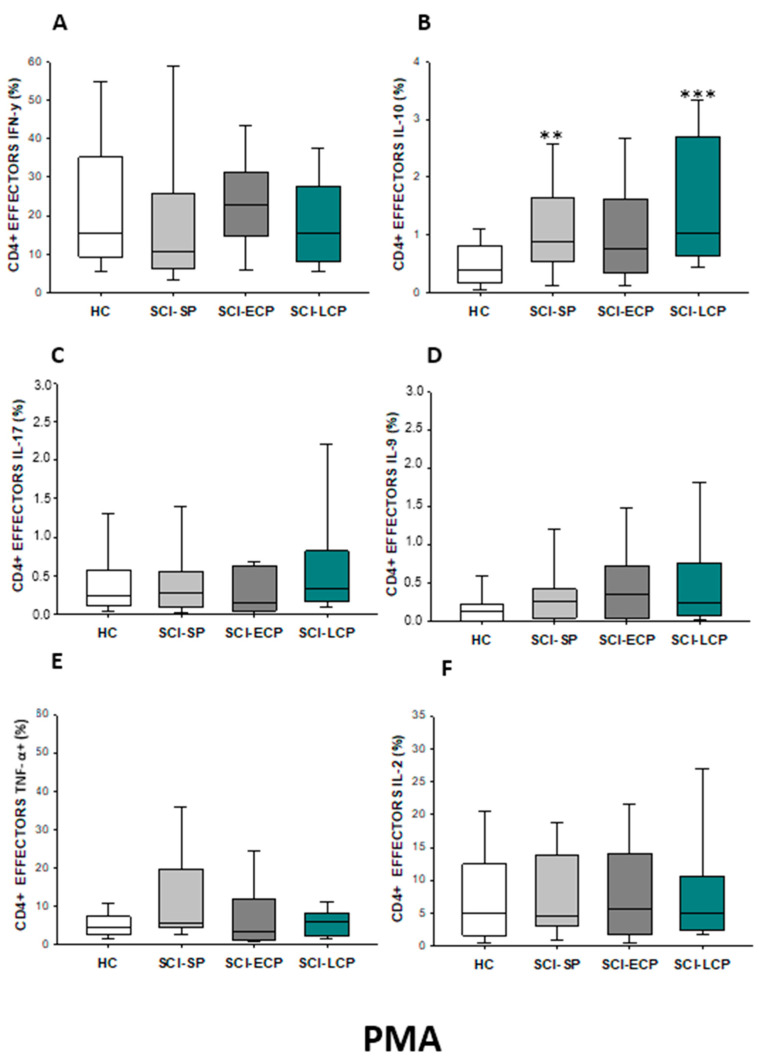
(**A**–**F**) Percentage of CD4 effector cells producing IFN-γ, IL-10, IL-17, IL-9, TNF-α, and IL-2 after PMA stimulation in healthy controls (HC); chronic SCI patients with short periods of evolution (<5 years) (SCI-SP); chronic SCI patients in early chronic phase (5 to 15 years) (SCI-ECP); and chronic SCI patients in late-chronic phase (>15 years) (SCI-LCP). We use ‘*’ to distinguish between chronic SCI patients and HC. *p* < 0.01 (**), and *p <* 0.001 (***).

Subsequently to PMA stimulation (Figure 6A–F), we show a significant increase in IL-10 production by CD4 effector cells in SCI-LCP versus HC (SCI-LCP = 1.03 [0.63–2.71]; HC = 0.395 [0.19–0.765], *** *p* = 0.001). Moreover, SCI-SP patients also exhibited higher production of IL-10 (SCI-SP = 0.875 [0.585–1.545], ** *p* = 0.01). Conversely, in this case, no significant differences between the SCI-SP and SCI-LCP groups were observed. 

#### 2.2.4. Cytokine Expression in CD4 Effector Memory Lymphocytes

**Figure 7:**
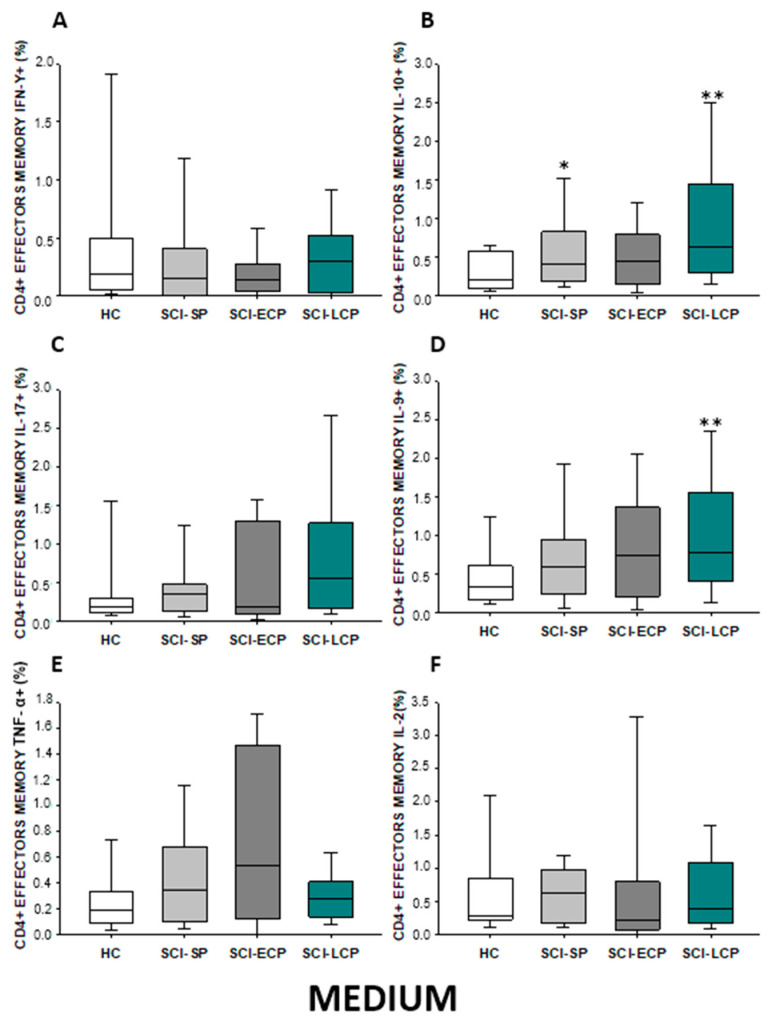
(**A**–**F**) Percentage of CD4 effector memory cells producing IFN-γ, IL-10, IL-17, IL-9, TNF-α, and IL-2 in healthy controls (HC); chronic SCI patients with short periods of evolution (<5 years) (SCI-SP); chronic SCI patients in early chronic phase (5 to 15 years) (SCI-ECP); and chronic SCI patients in late-chronic phase (>15 years) (SCI-LCP). We use ‘*’ to distinguish between chronic SCI patients and HC. *p* < 0.05 (*), *p* < 0.01 (**).

Regarding effector memory CD4 (Figure 7A–F), we were able to report a significant increase in IL-10 production in both SCI-LCP patients compared to their healthy controls (SCI-LCP = 0.64 [0.3–1.44]; HC = 0.21 [0.094–0.575], ** *p* = 0.002). We also observed a significant increase in this cytokine in SCI-SP patients (SCI-SP = 0.405 [0.215–0.82], * *p* = 0.046). Simultaneously, a significant rise in IL-9 production in the SCI-LCP group in comparison to HC was reported (SCI-LCP = 0.78 [0.41–1.56]; HC= 0.345 [0.165–0.585], ** *p* = 0.01).

**Figure 8:**
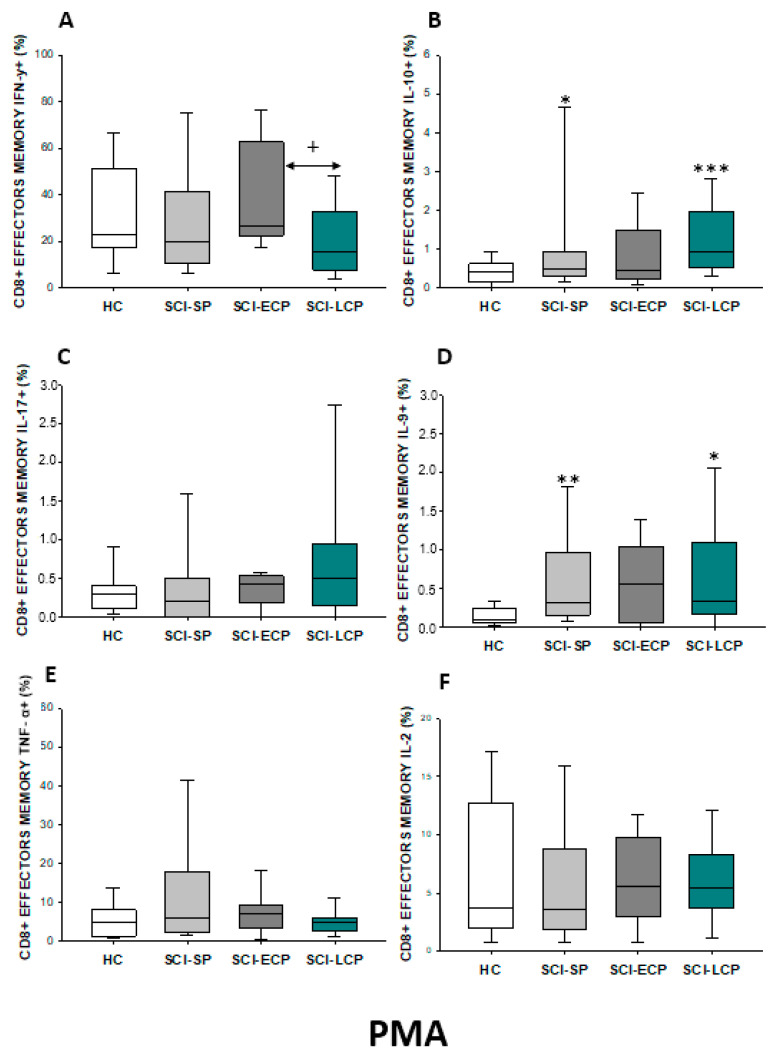
(**A**–**F**) Percentage of CD4 effector memory cells producing IFN-γ, IL-10, IL-17, IL-9, TNF-α, and IL-2 after PMA stimulation in healthy controls (HC); chronic SCI patients with short periods of evolution (<5 years) (SCI-SP); chronic SCI patients in early chronic phase (5 to 15 years) (SCI-ECP); and chronic SCI patients in late-chronic phase (>15 years) (SCI-LCP). We use ‘*’ to distinguish between chronic SCI patients and HC, whereas ‘+’ is used to compare chronic SCI patients. *p* < 0.05 (*/+), *p* < 0.01 (**), and *p <* 0.001 (***).

After PMA stimulation (Figure 8A–F), we define a significant increase in IL-10 production by effector memory CD4 cells in SCI-LCP versus HC (SCI-LCP = 1.240 [0.86–2.51]; HC = 0.65 [0.4–1], ** *p* = 0.003). Moreover, SCI-LCP patients also exhibited higher production of IL-9 (SCI-LCP = 0.34 [0.17–1.39]; HC = 0.17 [0.074–0.385], * *p* = 0.019). Likewise, enhanced production of IL-9 production by these cells in the SCI-ECP group was observed (SCI-ECP = 0.665 [0.15–1.23], * *p* = 0.031).

#### 2.2.5. Cytokine Expression in CD4 Central Memory Lymphocytes

**Figure 9:**
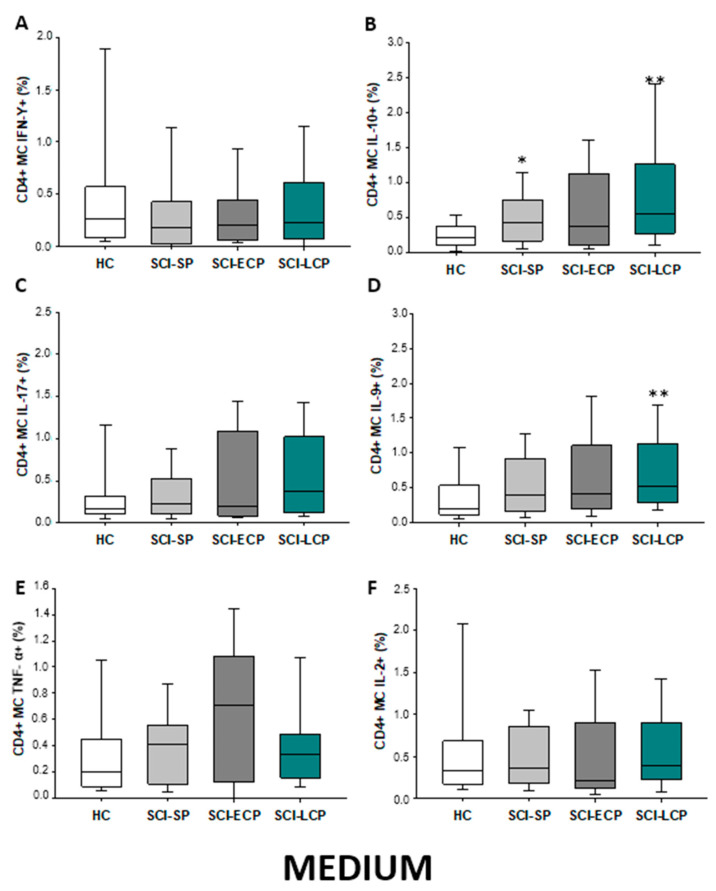
(**A**–**F**) Percentage of CD4 central memory cells producing IFN-γ, IL-10, IL-17, IL-9, TNF-α, and IL-2 in healthy controls (HC); chronic SCI patients with short periods of evolution (<5 years) (SCI-SP); chronic SCI patients in early chronic phase (5 to 15 years) (SCI-ECP); and chronic SCI patients in late-chronic phase (>15 years) (SCI-LCP). We use ‘*’ to distinguish between chronic SCI patients and HC. *p* < 0.05 (*), *p* < 0.01 (**).

Finally, for central memory CD4 (Figure 9A–F), we report that SCI-LCP patients, when compared to HC, displayed a significant increase in both IL-10 (SCI-LCP = 0.56 [0.28–1.26]; HC = 0.225 [0.118–0.37], ** *p* = 0.002) and IL-9 production (SCI-LCP = 0.81 [0.28–1.18]; HC = 0.21 [0.12–0.49], ** *p* = 0.01). Simultaneously, a significant rise in IL-10 was also observed in the SCI-SP group versus HC (SCI-SP = 0.435 [0.175–0.735], * *p* = 0.043).

**Figure 10:**
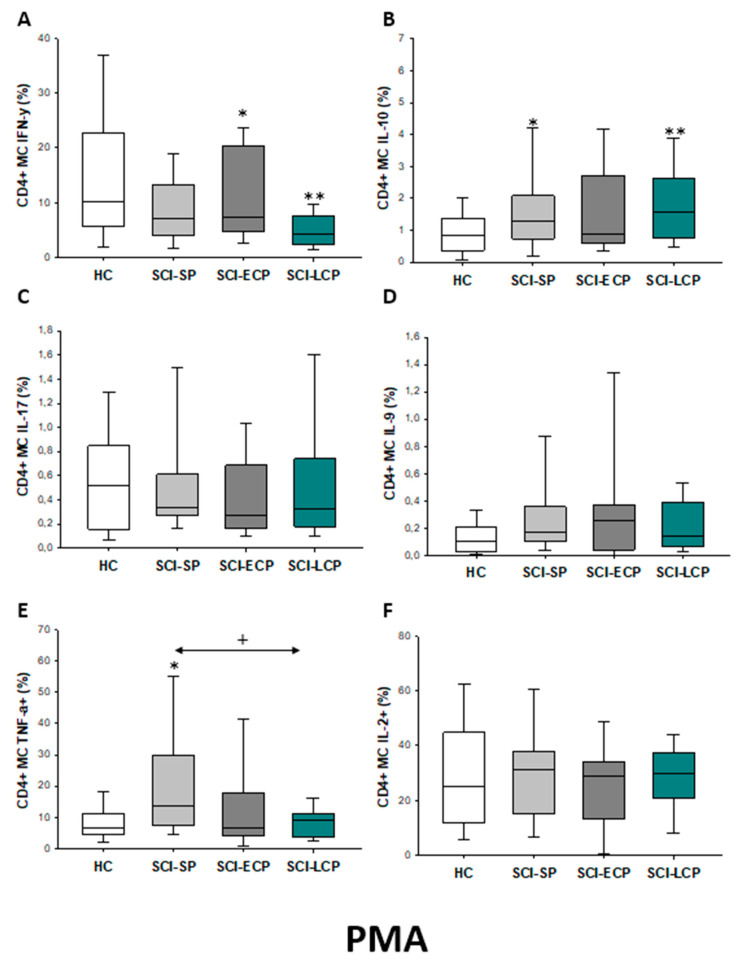
(**A**–**F**) Percentage of CD4 central memory cells producing IFN-γ, IL-10, IL-17, IL-9, TNF-α, and IL-2 after PMA stimulation in healthy controls (HC); chronic SCI patients with short periods of evolution (<5 years) (SCI-SP); chronic SCI patients in early chronic phase (5 to 15 years) (SCI-ECP); and chronic SCI patients in late-chronic phase (>15 years) (SCI-LCP). We use ‘*’ to distinguish between chronic SCI patients and HC, whereas ‘+’ is used to compare chronic SCI patients. *p* < 0.05 (*/+), *p* < 0.01 (**).

After PMA stimulation (Figure 10A–F), we could observe that, compared to controls, SCI-LCP subjects displayed a significant decrease in IFN-γ production (SCI-LCP = 4.38 [2.42–7.61]; HC = 10.17 [6.26–22.5], ** *p* = 0.004) together with an augmentation of IL-10 (SCI-LCP = 1.58 [0.77–2.61]; HC = 0.84 [0.365–1.32], ** *p* = 0.009). On the other hand, SCI-ECP patients equally presented a decrease in IFN-γ (SCI-SP = 7.45 [4.96–20.4], * *p* = 0.031). Finally, when compared to HC, SCI-SP patients showed an upregulation of IL-10 (SCI-SP = 1.295 [0.83–2.07], * *p* = 0.043) and TNF-α (SCI-SP = 13.5 [7.66–26.7]; HC = 6.73 [4.49–10.51], * *p* = 0.021). Importantly, the production of this cytokine was significantly higher in SCI-SP when we compared it to SCI-LCP subjects (SCI-LCP = 9.34 [3.88–11.1], * *p* = 0.032).

Overall, a summary of the results obtained can be found at the end of this section (Table 2).

### 2.3. Patients with Chronic Spinal Cord Injury Display a Distinct Cytokine Production by Circulating Total, Naïve, Effector, and Central/Effector Memory CD8 Cells Depending on the Years of Evolution

Next, we studied the IFN-γ, IL-10, IL-17, IL-9, TNF-α, and IL-2 expression by CD8 lymphocytes and their main subpopulations from SCI-SP, SCI-ECP, and SCI-LCP (*n* = 101) in comparison with HC.

#### 2.3.1. Cytokine Expression in CD8 Lymphocytes

**Figure 11:**
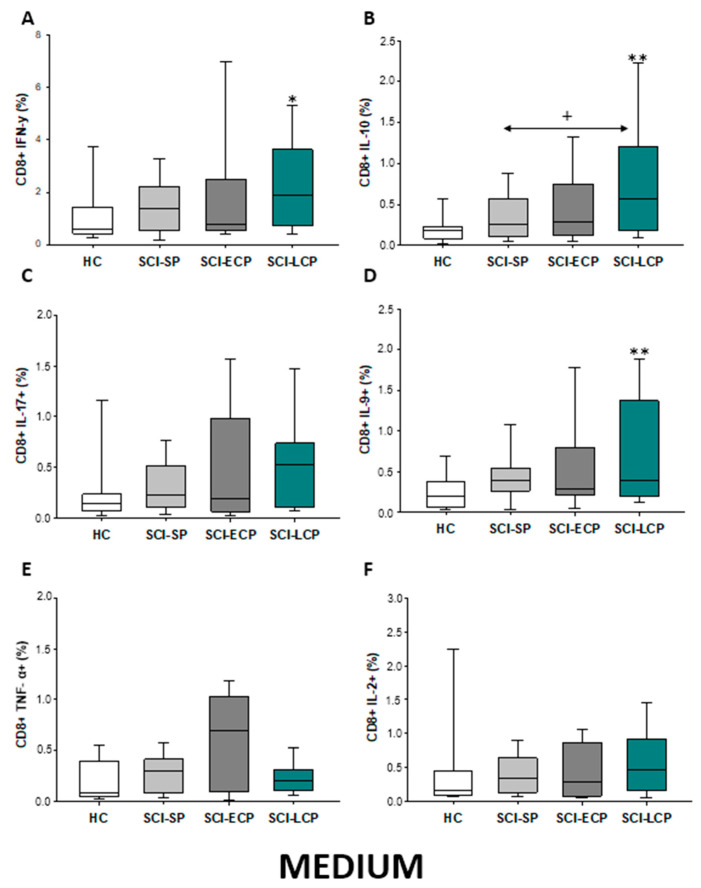
(**A**–**F**) Spontaneous cytokine expression by CD8 lymphocytes from SCI patients and HC. Percentage of CD8 cells producing IFN-γ, IL-10, IL-17, IL-9, TNF-α, and IL-2 in healthy controls (HC); chronic SCI patients with short periods of evolution (<5 years (SCI-SP); chronic SCI patients in early chronic phase (5 to 15 years) (SCI-ECP); and chronic SCI patients in late-chronic phase (>15 years) (SCI-LCP). We use ‘*’ to distinguish between chronic SCI patients and HC. *p* < 0.05 (*/+), *p* < 0.01 (**).

First, we analyzed the percentage of CD8 lymphocytes spontaneously producing IFN-γ, IL-10, IL-17, IL-9, TNF-α, and IL-2 (Figure 11A–F). Globally, our results define that, in comparison to HC, CD8 from patients with SCI-LCP display a significant increase in IFN-γ production (SCI-LCP = 1.87 [0.72–3.63]; HC = 0.575 [0.44–1.33], * *p* = 0.04), IL-10 (SCI-LCP = 0.57 [0.18–1.2]; HC = 0.185 [0.099–0.22], ** *p* = 0.002), and IL-9 (SCI-LCP = 0.4 [0.2–1.37]; HC = 0.205 [0.064–0.37], ** *p* = 0.01). In the event of IL-10, we also report that this increase was significantly higher in SCI-LCP versus SCI-SP patients (SCI-SP = 0.26 [0.109–0.525], * *p* = 0.032).

**Figure 12:**
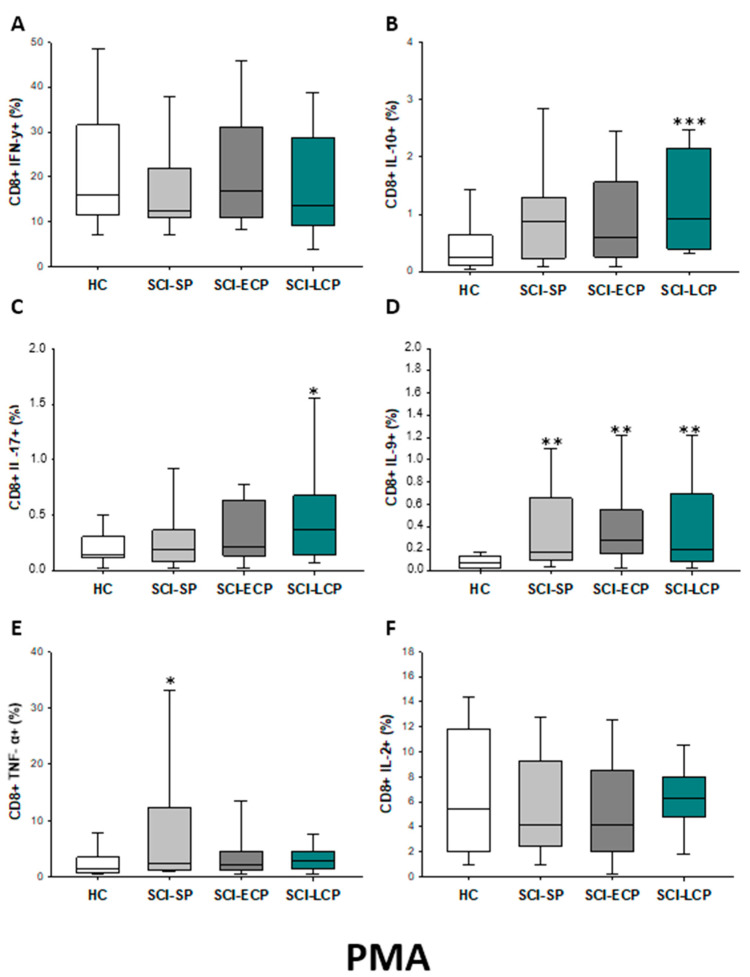
(**A**–**F**) Percentage of CD8 cells producing IFN-γ, IL-10, IL-17, IL-9, TNF-α, and IL-2 in healthy controls (HC) after PMA stimulation; chronic SCI patients with short periods of evolution (<5 years) (SCI-SP); chronic SCI patients in early chronic phase (5 to 15 years) (SCI-ECP); and chronic SCI patients in late-chronic phase (>15 years) (SCI-LCP). We use ‘*’ to distinguish between chronic SCI patients and HC. *p* < 0.05 (*), *p* < 0.01 (**), and *p <* 0.001 (***).

After PMA stimulation (Figure 12A–F), we observed that patients with SCI-LCP exhibit a significant increase in the release of IL-10 by CD8 cells in comparison to HC (SCI-LCP = 0.92 [0.39–2.15]; HC = 0.25 [0.105–0.605], *** *p* < 0.001); but also, in the production of IL-17 (SCI-LCP = 0.37 [0.15–0.68]; HC = 0.145 [0.125–0.28], * *p* = 0.028) and IL-9 (SCI-LCP = 0.2 [0.087–0.69]; HC = 0.071 [0.0305–0.125], ** *p* = 0.003). Regarding SCI-ECP, we only observed an increase in the production of IL-9 by CD8 cells (SCI-ECP = 0.275 [0.2–0.55], ** *p* = 0.002), whereas, for SCI-SP, we reported a significant increase in both IL-9 (SCI-SP = 0.175 [0.104–0.65], ** *p* = 0.002) and TNF-α (SCI-SP = 2.4 [1.3–10.55]; HC = 1.38 [0.75–3.09], * *p* = 0.032).

#### 2.3.2. Cytokine Expression in CD8 Naïve Lymphocytes

**Figure 13:**
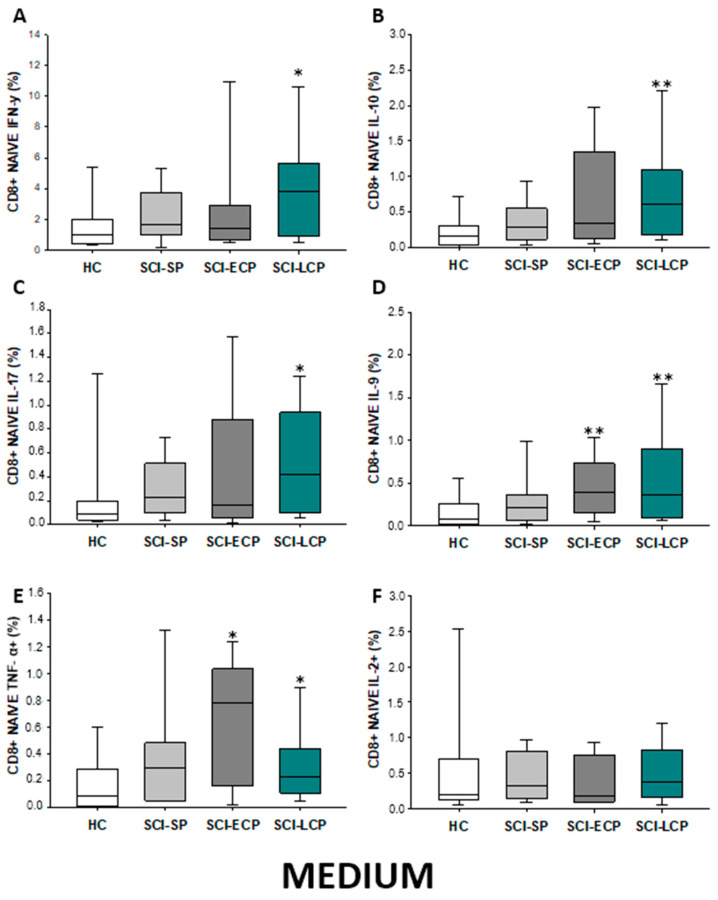
(**A**–**F**) Percentage of CD8 naïve cells producing IFN-γ, IL-10, IL-17, IL-9, TNF-α, and IL-2 in healthy controls (HC); chronic SCI patients with short periods of evolution (<5 years) (SCI-SP); chronic SCI patients in early chronic phase (5 to 15 years) (SCI-ECP); and chronic SCI patients in late-chronic phase (>15 years) (SCI-LCP). We use ‘*’ to distinguish between chronic SCI patients and HC. *p* < 0.05 (*), *p* < 0.01 (**).

In regard to CD8 naïve populations (Figure 13A–F), we could identify that when compared to HC, SCI-LCP patients displayed a significant increase in the production of IFN-γ (SCI-LCP = 3.83 [0.95–5.67]; HC = 1.03 [0.44–1.88], * *p* = 0.032), IL-10 (SCI-LCP = 0.61 [0.18–1.09]; HC = 0.17 [0.064–0.3], ** *p* = 0.002), IL-17 (SCI-LCP = 0.42 [0.098–0.94]; HC = 0.084 [0.04–0.19], * *p* = 0.02), IL-9 (SCI-LCP = 0.36 [0.1–0.9]; HC = 0.083 [0.025–0.23], ** *p* = 0.02), and TNF-α (SCI-LCP = 0.23 [0.11–0.44]; HC = 0.092 [0.014–0.28], * *p* = 0.032). Likewise, a significant increase was equally defined in SCI-ECP patients in terms of IL-9 (SCI-ECP = 0.395 [0.17–0.73], ** *p* = 0.006) and TNF-α production (SCI-ECP = 0.78 [0.29–1.035], * *p* = 0.017).

**Figure 14:**
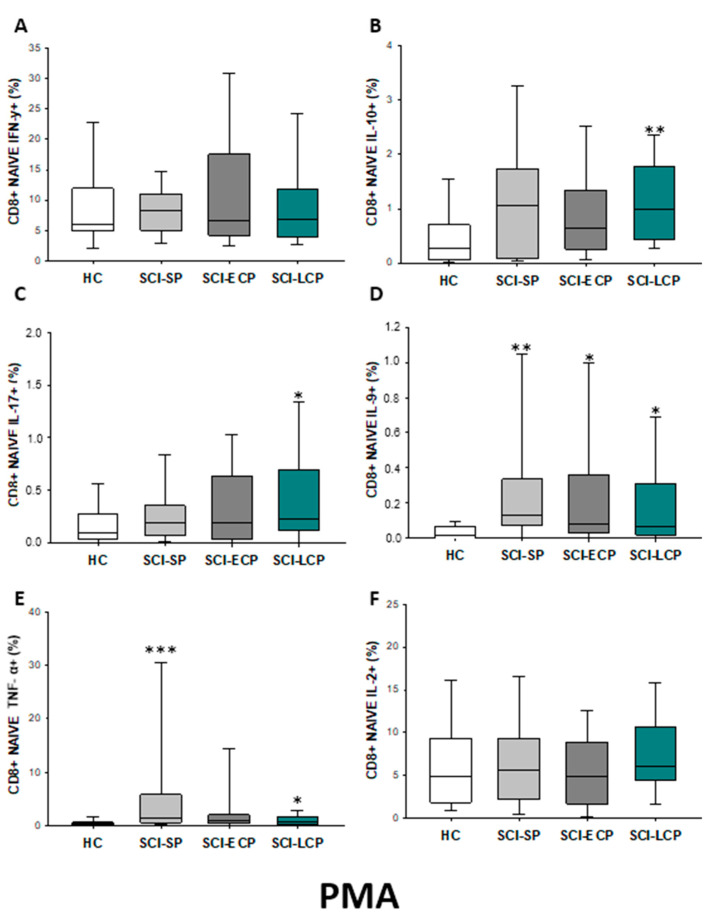
(**A**–**F**) Percentage of CD8 naïve cells producing IFN-γ, IL-10, IL-17, IL-9, TNF-α, and IL-2 in healthy controls (HC) after PMA stimulation; chronic SCI patients with short periods of evolution (<5 years) (SCI-SP); chronic SCI patients in early chronic phase (5 to 15 years) (SCI-ECP); and chronic SCI patients in late-chronic phase (>15 years) (SCI-LCP). We use ‘*’ to distinguish between chronic SCI patients and HC. *p* < 0.05 (*), *p* < 0.01 (**), and *p <* 0.001 (***).

Subsequently to PMA stimulation (Figure 14A–F), a significant increase in IL-10 production by CD8 naïve cells could be observed in SCI-LCP (SCI-LCP= 0.98 [0.42–1.77]; HC = 0.265 [0.097–0.68], ** *p* = 0.003) as well as in IL-17 (SCI-LCP= 0.23 [0.12–0.7]; HC = 0.094 [0.037–0.235], * *p* = 0.046), IL-9 (SCI-LCP = 0.065 [0.019–0.31]; HC = 0.017 [0–0.066], * *p* = 0.037); and TNF-α (SCI-LCP = 0.78 [0.37–1.75]; HC = 0.405 [0.210–0.675], * *p* = 0.03). Moreover, both SCI-ECP and SCI-SP patients exhibited higher production of IL-9 than HC (SCI-ECP = 0.082 [0.0320.35], * *p* = 0.023) (SCI-SP = 0.13 [0.079–0.315], ** *p* = 0.002), whereas SCI-SP subjects also displayed an enhanced production of TNF-α (SCI-SP = 1.51 [0.610–4.735] *** *p* < 0.001).

#### 2.3.3. Cytokine Expression in CD8 Effector Lymphocytes

**Figure 15:**
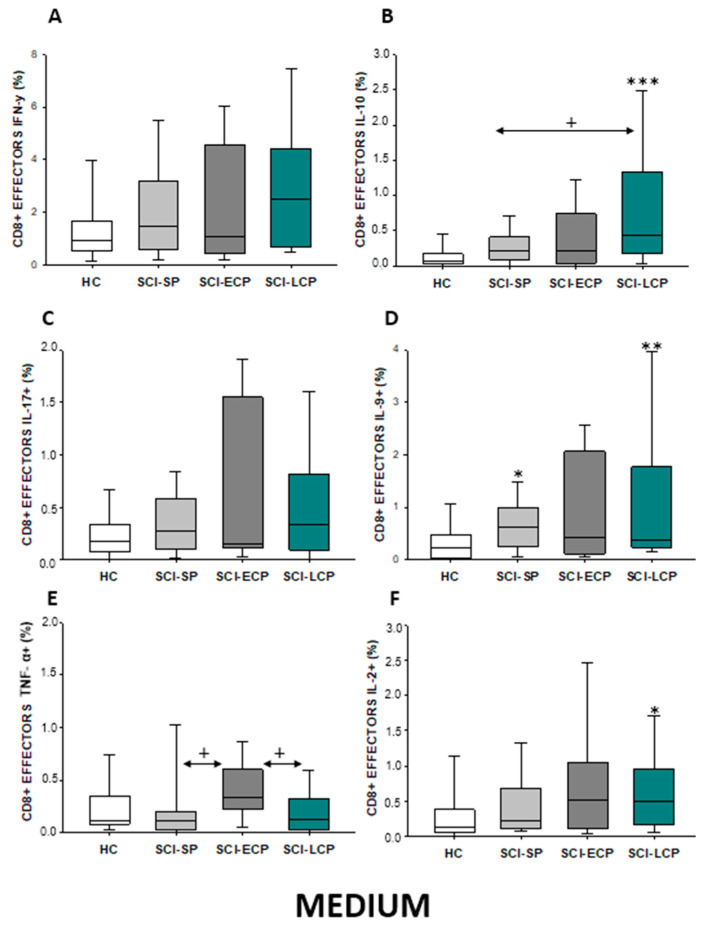
(**A**–**F**) Percentage of CD8 effector cells producing IFN-γ, IL-10, IL-17, IL-9, TNF-α, and IL-2 in healthy controls (HC); chronic SCI patients with short periods of evolution (<5 years) (SCI-SP); chronic SCI patients in early chronic phase (5 to 15 years) (SCI-ECP); and chronic SCI patients in late-chronic phase >15 years) (SCI-LCP). We use ‘*’ to distinguish between chronic SCI patients and HC, whereas ‘+’ is used to compare chronic SCI patients. *p* < 0.05 (*/+), *p* < 0.01 (**), and *p* < 0.001 (***).

In the event of effector CD8 lymphocytes (Figure 15A–F), our results show that there was a significant increase in IL-10 production observed in SCI-LCP patients when compared to healthy subjects (SCI-LCP = 0.43 [0.17–1.33]; HC = 0.067 [0.031–0.17], *** *p* = 0.01). Moreover, there is a similar increase in the production of this cytokine between SCI-LCP and SCI-SP (SCI-SP = 0.215 [0.095–0.385], + *p* = 0.026). In terms of IL-9 expression, we observed a significant increase in SCI-LCP patients (SCI-LCP = 0.37 [0.23–1.77]; HC = 0.22 [0.033–0.435], * *p* = 0.02) and SCI-SP (SCI-SP = 0.63 [0.265–0.97], * *p* = 0.032). Simultaneously, we observed a significant increase in TNF-α production in SCI-ECP patients when compared to SCI-LCP (SCI-ECP= 0.330 [0.290–0.51]; SCI-LCP = 0.130 [0.026–0.32], + *p* = 0.038) and SCI-SP (SCI-SP = 0.115 [0.034–0.200], + *p* = 0.027). Eventually, IL-2 production was significantly upregulated in the SCI-LCP group (SCI-LCP = 0.490 [0.17–0.96]; HC = 0.13 [0.064–0.365], * *p* = 0.037).

**Figure 16:**
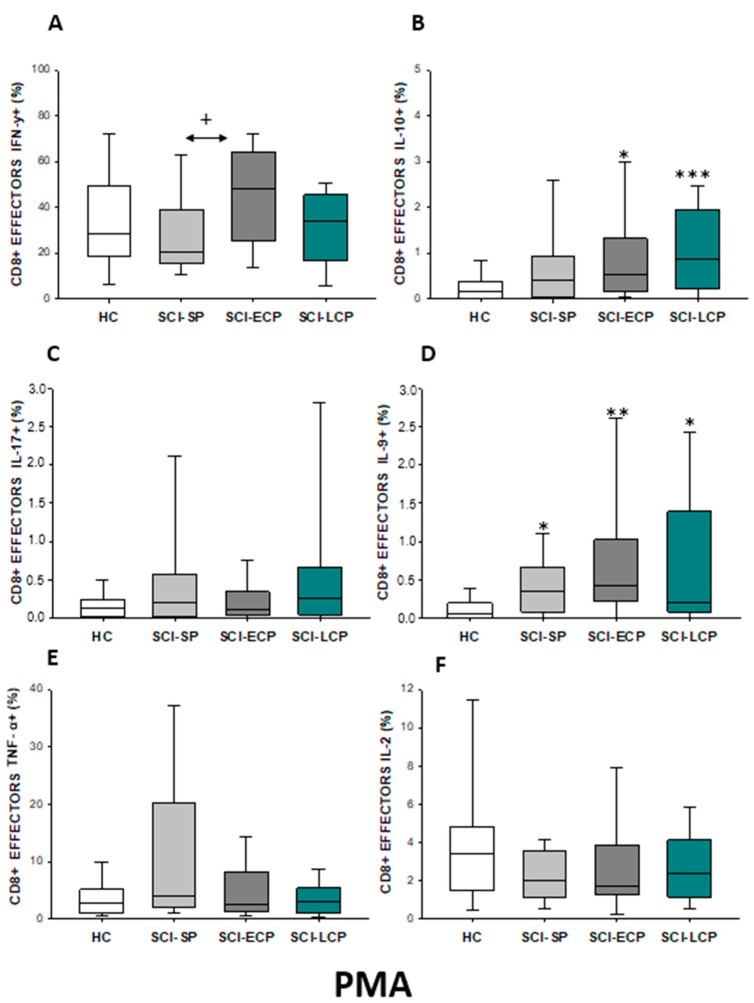
(**A**–**F**) Percentage of CD8 effector cells producing IFN-γ, IL-10, IL-17, IL-9, TNF-α, and IL-2 after PMA stimulation in healthy controls (HC); chronic SCI patients with short periods of evolution (<5 years) (SCI-SP); chronic SCI patients in early chronic phase (5 to 15 years) (SCI-ECP); and chronic SCI patients in late-chronic phase (>15 years) (SCI-LCP). We use ‘*’ to distinguish between chronic SCI patients and HC, whereas ‘+’ is used to compare chronic SCI patients. *p* < 0.05 (*/+), *p* < 0.01 (**), and *p <* 0.001 (***).

After PMA stimulation (Figure 16A–F), we defined a significant increase in IFN-γ production by effector CD8 cells in SCI-ECP when compared to SCI-SP (SCI-ECP = 48.3 [25.9–62]; SCI-SP = 20.65 [16.05–38.55], * *p* = 0.041). Simultaneously, in comparison to HC, we report a significant increase in IL-10 production in the SCI-LCP group (SCI-LCP = 0.86 [0.23–1.96]; HC = 0.165 [0.017–0.37], *** *p* = 0.001), which was equally observed in SCI-ECP patients (SCI-ECP = 0.515 [0.15–1.18], * *p* = 0.047). Lastly, compared to HC, we observed a significant increase in IL-9 production in all SCI patients, including SCI-LCP (SCI-LCP = 0.21 [0.082–1.39]; HC = 0.059 [0–0.018], ** *p* = 0.013), SCI-ECP (SCI-ECP = 0.415 [0.25–0.78], ** *p* = 0.003), and SCI-SP (SCI-SP = 0.345 [0.093–0.645], * *p* = 0.0017

#### 2.3.4. Cytokine Expression in CD8 Effector Memory Lymphocytes

**Figure 17:**
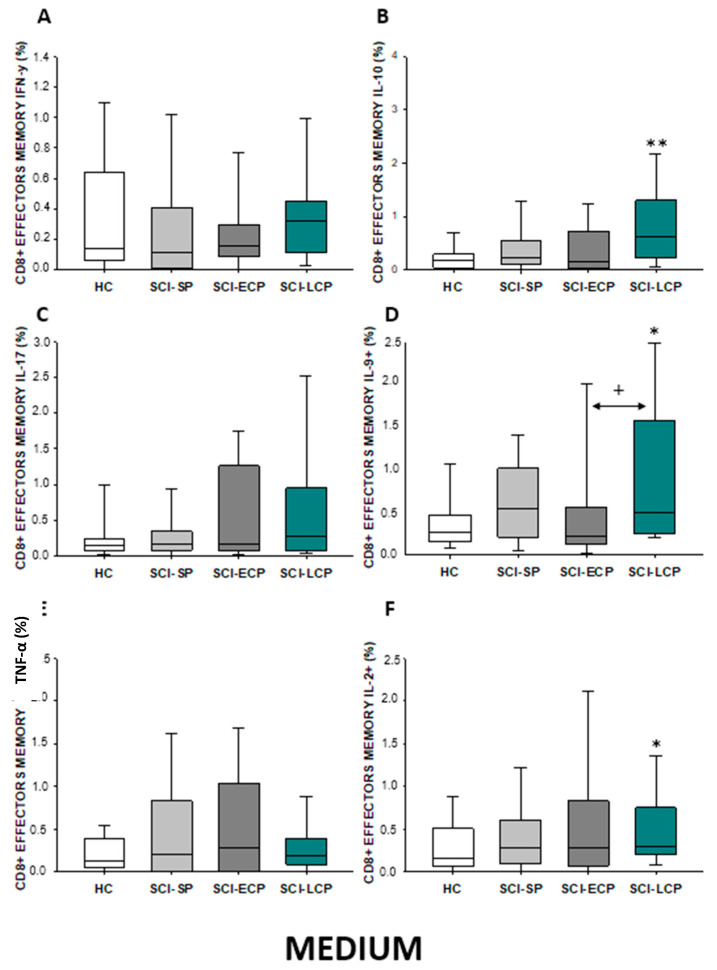
(**A**–**F**) Percentage of CD8 effector memory cells producing IFN-γ, IL-10, IL-17, IL-9, TNF-α, and IL-2 in healthy controls (HC); chronic SCI patients with short periods of evolution (<5 years) (SCI-SP); chronic SCI patients in early chronic phase (5 to 15 years) (SCI-ECP); and chronic SCI patients in late-chronic phase (>15 years) (SCI-LCP). We use ‘*’ to distinguish between chronic SCI patients and HC, whereas ‘+’ is used to compare chronic SCI patients. *p* < 0.05 (*/+), *p* < 0.01 (**).

On the other hand, we studied both effector and central memory CD8 lymphocytes in the different SCI subgroups. Regarding effector memory CD8 (Figure 17A–F), we were able to report a significant increase in IL-10 production in SCI-LCP patients compared to their healthy controls (SCI-LCP = 0.62 [0.23–1.3]; HC = 0.175 [0.046–0.285], ** *p* = 0.003). Simultaneously, a significant increase in IL-9 production in the SCI-LCP group was reported in comparison to the HC (SCI-LCP = 0.5 [0.25–1.58]; HC = 0.26 [0.165–0.465], * *p* = 0.02) and with the SCI-ECP group (SCI-ECP = 0.225 [0.12–0.47], + *p* = 0.026). Eventually, a significant increase in IL-2 production by effector memory CD8 cells was reported in the SCI-LCP group (SCI-LCP = 0.29 [0.2–0.75]; HC = 0.16 [0.087–0.49], * *p* = 0.043).

**Figure 18:**
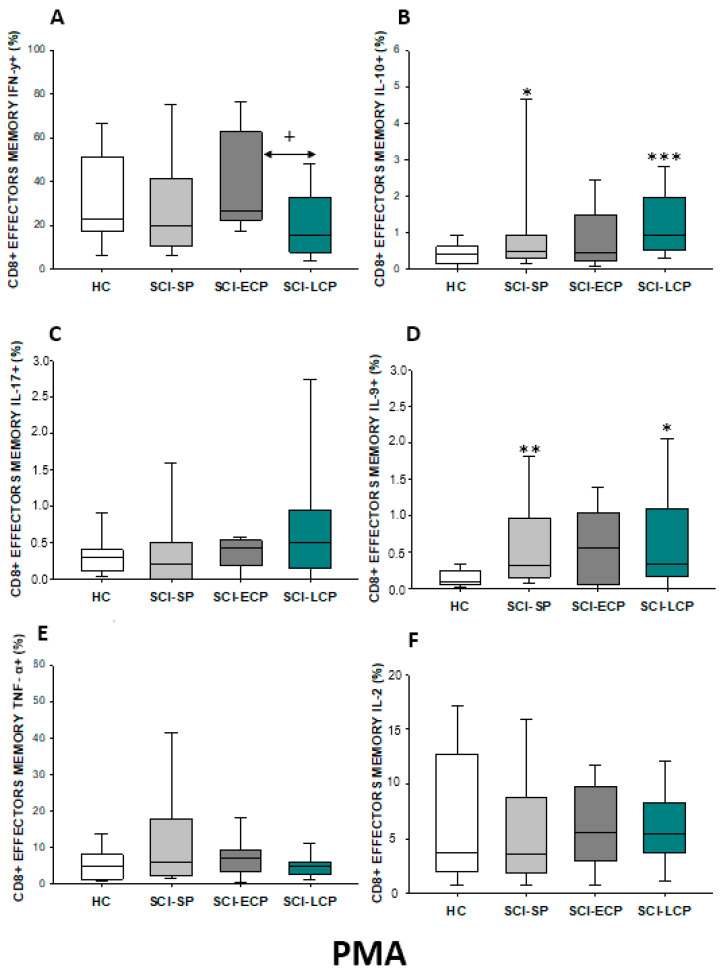
(**A**–**F**) Percentage of CD8 effector memory cells producing IFN-γ, IL-10, IL-17, IL-9, TNF-α, and IL-2 after PMA stimulation in healthy controls (HC); chronic SCI patients with short periods of evolution (<5 years) (SCI-SP); chronic SCI patients in early chronic phase (5 to 15 years) (SCI-ECP); and chronic SCI patients in late-chronic phase (>15 years) (SCI-LCP). We use ‘*’ to distinguish between chronic SCI patients and HC, whereas ‘+’ is used to compare chronic SCI patients. *p* < 0.05 (*/+), *p* < 0.01 (**), and *p <* 0.001 (***).

After PMA stimulation (Figure 18A–F), we observed a significant decrease in IFN-γ production in the SCI-LCP when compared to the SCI-ECP group (SCI-LCP = 15.8 [7.74–32.8]; SCI-ECP = 26.35 [22.6–61.7], + *p* = 0.02). Concomitantly, we observed a noteworthy increase in IL-10 production in the SCI-LCP group (SCI-LCP = 0.94 [0.53–1.97]; HC = 0.4 [0.16–0.58], *** *p* = 0.001). Moreover, when compared to HC, increased production of IL-9 was observed in SCI-LCP (SCI-LCP = 0.33 [0.17–1.1]; HC = 0.096 [0.059–0.245], * *p* = 0.015) and in the SCI-SP group (SCI-SP = 0.315 [0.165–0.965], ** *p* = 0.002).

#### 2.3.5. Cytokine Expression in CD8 Central Memory Lymphocytes

**Figure 19:**
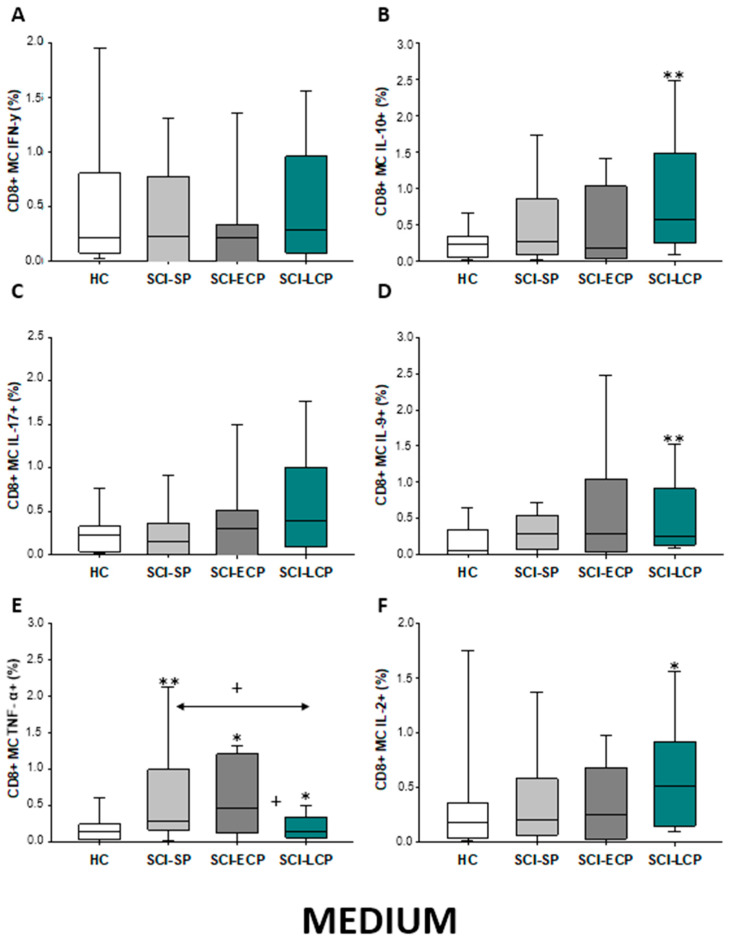
(**A**–**F**) Percentage of CD8 memory central cells producing IFN-γ, IL-10, IL-17, IL-9, TNF-α, and IL-2 in healthy controls (HC); chronic SCI patients with short periods of evolution (<5 years) (SCI-SP); chronic SCI patients in early chronic phase (5 to 15 years) (SCI-ECP) and chronic SCI patients in late-chronic phase (>15 years) (SCI-LCP). We use ‘*’ to distinguish between chronic SCI patients and HC, whereas ‘+’ is used to compare chronic SCI patients. *p* < 0.05 (*/+) and *p* < 0.01 (**).

Finally, we studied cytokine production by CD8 memory cells (Figure 19A–F), reporting that compared to HC, SCI-LCP patients tend to show increased IL-10 (SCI-LCP = 0.57 [0.25–1.49]; HC = 0.24 [0.067–0.345], ** *p* = 0.002), IL-9 (SCI-LCP = 0.26 [0.14–0.91]; HC = 0.067 [0.015–0.34], ** *p* = 0.002), TNF-α (SCI-LCP = 0.140 [0.064–0.35]; HC = 0.15 [0.045–0.245], * *p* = 0.042), and IL-2 (SCI-LCP = 0.51 [0.15–0.92]; HC = 0.18 [0.045–0.325], * *p* = 0.032). On the other hand, SCI-ECP displayed a remarkable increase only in TNF-α production (SCI-ECP = 0.47 [0.18–1.13], * *p* = 0.032). Moreover, the same cytokine was increased in SCI-SP patients (SCI-SP = 0.295 [0.185–0.990], ** *p* = 0.01), and we equally observe that this increase is significantly higher than observed in the SCI-LCP group (* *p* = 0.034).

**Figure 20:**
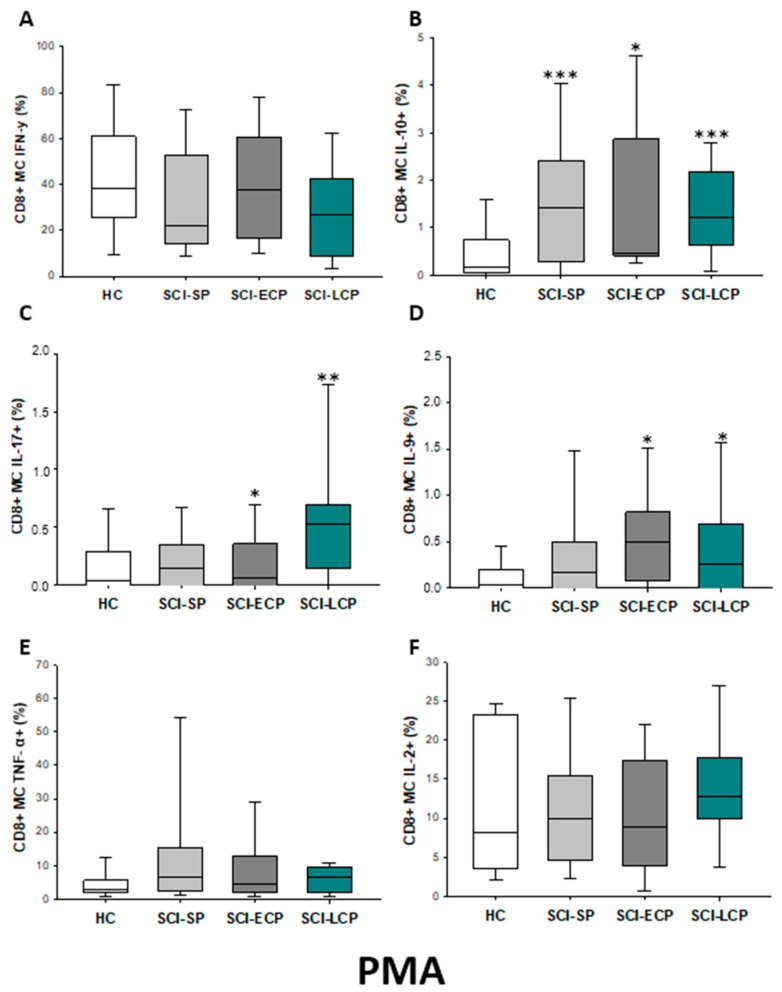
(**A**–**F**) Percentage of CD8 memory central cells producing IFN-γ, IL-10, IL-17, IL-9, TNF-α, and IL-2 after PMA stimulation in healthy controls (HC); chronic SCI patients with short periods of evolution (<5 years) (SCI-SP); chronic SCI patients in early chronic phase (5 to 15 years) (SCI-ECP); and chronic SCI patients in late-chronic phase (>15 years) (SCI-LCP). We use ‘*’ to distinguish between chronic SCI patients and HC, whereas ‘+’ is used to compare chronic SCI patients. *p* < 0.05 (*), *p* < 0.01 (**), and *p <* 0.001 (***).

After PMA stimulation (Figure 20A–F), we report that CD8 memory cells of SCI-LCP presented higher IL-10 production (SCI-LCP = 1.23 [0.64–2.17]; HC = 0.16 [0.052–0.71], *** *p* = 0.001), IL-17 (SCI-LCP = 0.53 [0.15–0.7]; HC= 0.043 [0–0.29], ** *p* = 0.01) and IL-9 (SCI-LCP = 0.26 [0–0.69]; HC = 0.03 [0–0.2], * *p* = 0.035). Concomitantly, the SCI-ECP group showed a similar increase in IL-10 (SCI-ECP = 0.47 [0.41–2.86], * *p* = 0.014), IL-17 (SCI-ECP = 0.06 [0–0.36], * *p* = 0.031) and IL-9 (SCI-ECP = 0.49 [0.11–0.82], * *p* = 0.031), whereas for SCI-SP we only observed a significant increase in IL-10 production (SCI-SP = 1.425 [0.305–2.345], *** *p* = 0.001). 

Overall, a summary of the results obtained can be found at the end of this section (Table 3).

## 3. Discussion

In recent years, great progress has been made in the survival of patients who have suffered an SCI. The long duration of chronic SCI has shown that patients experience different events that complicate their quality of life in addition to established neurological deficits [28]. The high incidence of infectious events as well as the development of systemic complications, contribute to the deterioration of their quality of life [15,29]. Understanding the pathogenic mechanisms that condition the evolution of patients with chronic SCI is a critical objective in trying to carry out therapeutic interventions for improvement. There is increasing evidence of impaired immune systems and T lymphocytes in patients with chronic SCI [30,31]. In this work, we have investigated the pattern of cytokine secretion, focused on IFN-γ, IL-10, IL-17, IL-9, TNF-α, and IL-2 expression by CD4 and CD8 T lymphocytes in a large population of patients with chronic SCI stratified for periods of evolution after the acute spinal event. We have found that we have shown that the pattern of alteration of cytokine secretion is different throughout the evolution of patients.

Firstly, we have observed a differential expansion of CD4 and CD8 lymphocytes that express IL-10 in patients with SCI-LCP. Interestingly, this overexpression is observed in both stimulated and unstimulated CD4 and CD8 lymphocytes and in all their different stages of activation/differentiation. IL-10 modulates the inflammatory response, decreasing the production of some proinflammatory cytokines and inhibiting antigen presentation while improving its uptake and clearance functions [32]. Increased IL-10 expression is associated with the mechanisms of suppression of inflammatory states [32]. In addition, high IL-10 expression is associated with a predisposition and vulnerability to infections [33,34]. However, in chronic stages, this molecule can be an indicator of a hyperactivated immunoinflammatory response, appearing as a mechanism to guarantee the protection of a host from excessively exuberant responses to pathogens and microbiota [35]. In this sense, patients with SCI tend to suffer from more infectious processes, especially skin and soft tissue infections (SSTI), UTIs, and bloodstream infections [29]. Likewise, there is established evidence that patients with chronic SCI exhibit an altered intestinal barrier, gut dysbiosis, and enhanced bacterial translocation, leading to an increase in different proinflammatory cytokines [18]. Hence, the enhanced CD4 and CD8 lymphocyte production of IL-10 in patients with SCI-LCP could be the result of persistent pathogens and bacterial antigen exposure. Thus, a vicious circle of IL-10 stimulation by microorganisms and a defective protective response against infections may be suggested.

We have also observed a spontaneous and stimulated overexpression of IL-9 by CD4 and C8 lymphocytes at different stages of activation/differentiation in patients with SCI-LCP. In contrast to the IL-10 observed pattern of expression, stimulated CD8 lymphocytes from patients with SCI-SP and SCI-ECP also show increased levels of IL-9 after stimulations. IL-9 is a pleiotropic cytokine that has been involved in response to infectious agents and in the pathogenesis of autoimmune inflammatory disorders of the central nervous system [36]. Interestingly, IL-9 appears to be overproduced in acute phases of SCI [37]. Thus, it is possible to claim a potential pathogenic role of IL-9 in SCI-LCP, although further work is needed in this sense.

TNFα expression by chronic CD4 and CD8 lymphocytes from chronic SCI show a different pattern to those observed for IL-10 and IL-9. TNFα overexpression is observed at different stages of CD8 activation/differentiation in patients in all three phases of the clinical course. In contrast, increased TNFα production by CD4 lymphocytes is found in SCI SCP. TNFα is a key regulatory and effector cytokine [38]. It has been proposed that this cytokine may play a dual role in the pathogenesis of SCI, mediating degenerative and reparative mechanisms, and its role probably depends on the stage of SCI and the cell population it influences [39]. Therefore, peripheral TNFα blockade has not brought any therapeutic benefit in animal models after SCI, which supports the need to delve into the role of this cytokine in this chronic condition. In our study, we have observed variable and dynamic spontaneous or stimulated overexpression and overproduction of TNFα at different stages and activation/differentiation of CD8 lymphocytes. This finding suggests that this exaggerated production is reactive to variable stimuli throughout the evolution of the patient with SCI. Some studies have reported that elevated levels of TNF-α and a suite of proinflammatory cytokines may be observed by patients in patients with chronic SCI, especially in patients with pain, UTIs, and pressure ulcers [40,41]. On the other hand, previous studies have noted that patients with chronic SCI tend to exhibit reduced levels of TNFα than HCs, especially those presenting with accelerated immunosenescence after repeated urinary tract infections [13]. We have observed that TNFα expression by central memory CD8 is decreased in SCI-LCP with respect to SCI-SP, which might indicate a depletion of these populations.

We observed a localized pattern of IFNγ overexpression preferentially focused on unstimulated naïve CD8 lymphocytes from SCI-LCP. The precise role of IFNγ in chronic SCI is not fully understood yet [42]. In patients with chronic SCI, IFNγ may be related to enhanced cytotoxic actions of activated CD8 cells, as IL-10 appears to boost the production of this cytokine along with other cytolytic factors, such as granzyme B [35]. Simultaneously, we report a marked downregulation of IFNγ released by CD4 memory cells from SCI-LCP patients. In agreement with our results, Zha et al. [25] also observed a downregulation of IFNγ production by CD4 T lymphocytes in response to PMA stimulation by chronic SCI and concluded that T cell exhaustion contributes to SCI-induced T cell dysfunction.

Finally, we have also found a variable increased expression of IL-17 in naïve or central memory CD4 and CD8 lymphocytes from CSI EFP and SCI-LCP. IL-17 has been shown to be involved in neuroinflammatory processes, being related in vivo to severe stages of the disease [43]. Furthermore, IL-17 seems to negatively modulate the regenerative processes of ependymal cells after SCI, impairing recovery [44]. Future studies should be aimed at evaluating the potential pathogenic role and potential targeting of IL-17 inhibitors in patients with SCI-LCP.

Overall, our work demonstrates an abnormal pattern of cytokine secretion by CD4 and CD8 lymphocytes in patients with SCI that varies throughout the stages of disease evolution. In addition, this T lymphocytic dysfunction tends to increase in patients with SCI-LCP, those with a higher time of evolution. These alterations confirm a state of dysregulation of the immune response with the possible involvement in the pathogenesis of the systemic complications of chronic SCI. The study of the cause of this T lymphocyte dysfunction is not the object of this work. However, it should be kept in mind that it is not related to the age or sex of the different groups of patients and controls. Furthermore, the T cell abnormalities are not explained by the impact of potential comorbidities. The exclusion criteria applied to the patients prevented the inclusion of those who suffered from serious diseases that could affect the immune system. In relation to the potential participation of infections in the induction of T lymphocyte alterations, patients with chronic infections and those who had suffered an acute infection in the previous three months were excluded. However, it is possible to suggest that some patients had more episodes of recurrent acute infections prior to inclusion in the study.

It should be noted that our findings show the evolutionary variability of at least the T compartment alterations with the years of evolution of chronic SCI, which can help us to understand discrepant results in relation to the study of the immune-inflammatory response of these patients. On the other hand, our work highlights the relevance of carrying out prospective follow-up studies for years in patients with chronic SCI and the potentiality of carrying out therapeutic immunointervention.

## 4. Materials and Methods

### 4.1. Study Design

We performed a prospective study on 105 patients with chronic SCI. To properly study the immune system through the course of SCI, patients were divided into three subpopulations: SCI-SP (1–5 years post-injury); SCI-ECP (5–15 years post-injury); and SCI-LCP (>15 years). Concomitantly, SCI subjects were compared with sex and age-matched healthy controls (HC). All participants were properly informed and provided their signed written consent approved by the Institutional Review of National Hospital for Paraplegic Patients (10 September 2015). The present research was completed following the basic ethical principles of autonomy, beneficence, non-maleficence, and distributive justice, following the 161 statements of Good Clinical Practice, the principles contained in the most recent Declaration of Helsinki (2013) and the Oviedo Convention (1997). The data and 163 pieces of information collected followed current legislation on data protection (Organic Law 164 3/2018 of 5 December, Protection of Personal Data and Guarantee of Digital Rights and 165 Regulation (EU) 2016/679)

Medical information from the SCI patients was collected in a routine clinical examination in the Physical Medicine and Rehabilitation Department, including the following data: (1) baseline demographic features; (2) time and mechanism of injury; (3) neurologic injury level and related severity; (4) tonic and phasic spasticity; (5) presence or absence, of pain, its type and seriousness; (6) history of past infections and other indicators of a chronic SCI complication; (7) comorbidities; (8) use of contemporary medications; (9) fatigue; (10) anxiety and depression levels; (11) degree of independence in daily living activities; and (12) self-reported quality of life and health status.

Blood samples were extracted from all individuals via standard venipuncture by an established aseptic technique. Samples were obtained from chronic SCI patients at the time of the medical evaluation in the outpatient clinic area. The inclusion criteria considered in this study were: (1) being ≥18 years; (2) history of SCI in a period ≥1 year, occurring at any level; and (3) SCI with varied severity, ranging from grades A to E according to the American Spinal Injury Association (ASIA) Impairment Scale (AIS) [45]. A Physical and Rehabilitation Medicine board-certified clinician in SCI medicine evaluated the subjects’ injuries according to the International Standards for Neurologic Classification of Spinal Cord Injury [46,47]. Conversely, exclusion criteria were as follows: (1) a coincident infection with notable severity, such as urinary tract infection 146 (UTI) or a respiratory infection, evidenced with a positive culture in the last 3 months; (2) chronic viral or bacterial infection; (3) clinical diagnosis of autoimmune disease; (4) serious cardiovascular disease (CVD); (5) hematopoietic, renal, lung or hepatic complications; (6) an endocrine or metabolic disorder, (i.e., type 1/2 diabetes mellitus); (7) previous history of cancer; (8) pressure ulcers in the last year; (9) administration of immunomodulatory drugs such as steroids in the last 3 months; (10) suffering from immunodeficiency or malnutrition; (11) being in pregnancy or lactation period; and (12) having undergone any psychiatric disorder.

### 4.2. Isolation of Peripheral Blood Mononuclear Cells

Peripheral blood mononuclear cells (PBMCs) were isolated by using the Ficoll Hypaque (LymphoprepTM, Axis-Shield, Oslo, Norway) gradient centrifugation. Then, cells were resuspended in RPMI 1640 (BioWhittaker Products, Verviers, Belgium) supplemented with 10% heat-inactivated fetal calf serum, 25 mM HEPES (BioWhittaker Products) and 1% penicillin–streptomycin (BioWhittaker Products). Cell enumeration was performed by conventional light microscopy in a Neubauer chamber following the trypan blue dead cells exclusion criteria. The viability of PBMCs was assessed by both trypan blue (light microscopy) and 7-aminoactinomycin D (7-AAD) (flow cytometry) exclusion.

### 4.3. Immunophenotype and Intracellular Cytokines Studies

One million and a half PBMCs were incubated with 1 µM of monensine (Sigma, St. Louis, MO, USA), 1 µg/mL of phorbol 12-myristate 13-acetate (PMA) (Sigma, MO, USA), and 1 µg/mL of ionomicine(Merk Milipore, Burlington, MA, USA). PBMCs were incubated at 37 °C, 5% CO_2_ for 6 h in a 15 mL tube. The cells were labeled with CD45RA-APC, CD3-Alexa 700, CCR7-c-PE-Cy7 (Beckton Dickinson, Franklin Lakes, NJ, USA) CD27-APC-Alexa 780, CD8-Alexa 405, and excluding vital Acqua-QD565 (Invitrogen, Waltham, MA, USA) monoclonal antibodies. For intracytoplasmic staining, cells were fixed and permeabilized with Fix and Perm kit (Caltag Laboratories, Burlingame, CA, USA), and cytokines stained with IL-17A-Alexa 488, IL-2-FITC, IL-9-PerCP-Cy 5.5, IFN-γ-Alexa 700 (Beckton Dickinson, NJ, USA) and IL-10-PE (Biolegend, San Diego, CA, USA) monoclonal antibodies. PBMCs were incubated at dark and 4 °C for about 20 min. Then washed with PBS 1X and incubated with solution A of Fix and Perm kit (Caltag Laboratories) for 15 min at dark and room temperatures as per manufacturer recommendations. Cells were washed again, and solution B of Fix and Perm kit was added. For intracytoplasmic staining, cytokines stained with IL-17A-Alexa 488, IL-2-FITC, IL-9-PerCP-Cy 5.5, IFN-γ-Alexa 700 (Beckton Dickinson, NJ, USA) and IL-10-PE (Biolegend, CA, USA) monoclonal antibodies were used. After a final wash, cells were resuspended in 100 µL of PBS 1× for the posterior acquisition in a BDFACSAria II flow cytometer (Beckton Dickinson, NJ, USA).

### 4.4. Statistical Analysis

Nonparametric Mann–Whitney U tests were applicated to compare chronic SCI patients and HC. All calculations were carried out with the Statistical Package for the Social Sciences (SPSS, version 22.0, Chicago, IL, USA). The data are expressed as the median with interquartile range (IQR). Significance was established at *p*-values (*p*) < 0.05 (*), *p* < 0.01 (**), and *p <* 0.001 (***).

## 5. Conclusions

Our study demonstrates an altered profile of cytokine-producer T cells in patients with chronic SCI. In addition, notable changes are also observed throughout the disease. In more detail, we have observed significant variations in cytokine production by circulating naive, effector, and memory CD4 and CD8 T cells. An exacerbated CD4/CD8 T cell production of IL-10 and IL-9 are the most notable findings in our study, especially in patients with SCI-LCP. However, changes in other cytokines such as IL-17, TNF-α, and IFN-γ also seem to be relevant changes in the immunological profile of these patients, and future studies should be directed to study the possible clinical consequences or develop additional translational approaches.

## Figures and Tables

**Table 1:** Demographic data of healthy controls (HC) and SCI patients.

Variable	HC (*n* = 38)	SCI (*n* = 105)	SCI-SP (*n* = 31)	SCI-ECP (*n* = 32)	SCI-LCP (*n* = 42)
Age (years)	25.41 ± 7.99	36.33 ± 13.24	29.24 ± 14.40	36.81 ± 12.26	40.28 ± 17.65
Sex (men/women)	42.10%/57.90%	74.28%/25.72%	90.32%/9.68%	77.78%/22.22%	61.70%/38.30%
Time of injury (years)		13.24 ± 9.47	2.30 ± 1.54	10.11 ± 2.55	22.26 ± 5.33
ASIA					
A		46.67%	41.93%	55.55%	44.68%
B		16.19%	3.70%	3.70%	21.28%
C		16.19%	18.51%	18.52%	19.15%
D		20.95%	22.22%	22.22%	14.89%
Injury level					
C1–C4		23.80%	22.22%	22.22%	14.89%
C5–C8		20.00%	18.51%	18.52%	25.53%
T1–T6		26.27%	25.92%	25.93%	29.79%
T7–T12		20.95%	29.62%	29.63%	14.89%
L1–L6		8.57%	3.70%	3.70%	14.89%

**Table 2:** A summary of the cytokines produced by CD4 lymphocytes and their main subpopulations in basal conditions (medium) and after PMA stimulation. ↑ = Increased in our study; ↓ = decreased in our study; ___ = No variation.

	CD4 Lymphocytes		CD4 Naïve		CD4 Effector		CD4 Effector Memory		CD4 Central Memory	
	*Medium*	*PMA*	*Medium*	*PMA*	*Medium*	*PMA*	*Medium*	*PMA*	*Medium*	*PMA*
**IFN-γ**	__________	__________	__________	__________	__________	__________	__________	__________	__________	**↓** in SCI-LCP and SCI-ECP versus HC
**IL-10**	**↑** in SCI-LCP when compared to HC and SCI-SP	**↑** in SCI-LCP and SCI-SP when compared to HC	**↑** in SCI-LCP when compared to HC	**↑** in SCI-LCP and SCI-SP when compared to HC	**↑** in SCI-LCP when compared to HC and SCI-SP (also **↑** relative to HC)	**↑** in SCI-LCP and SCI-SP when compared to HC	**↑** in SCI-LCP and SCI-SP when compared to HC	**↑** in SCI-LCP when compared to HC	**↑** in SCI-LCP and SCI-SP when compared to HC	**↑** in SCI-LCP and SCI-SP when compared to HC
**IL-17**	__________	__________	**↑** in SCI-LCP and SCI-ECP when compared to HC	__________	__________	__________	__________	__________	__________	__________
**IL-9**	**↑** in SCI-LCP when compared to HC	**↑** in SCI-LCP when compared to HC	**↑** in SCI-LCP and SCI-ECP when compared to HC	**↑** in SCI-LCP when compared to HC	__________	__________	**↑** in SCI-LCP when compared to HC	**↑** in SCI-LCP and SCI-ECP when compared to HC	**↑** in SCI-LCP when compared to HC	__________
**TNF-α**	**↑** in SCI-SP when compared to HC	**↑** in SCI-SP when compared to HC	__________	**↑** in SCI-SP when compared to HC	__________	__________	__________	__________	__________	**↑** in SCI-SP when compared to HC and with SCI-LCP
**IL-2**	__________	__________	__________	__________	__________	__________	__________	__________	__________	__________

**Table 3:** A summary of the cytokines produced by CD8 lymphocytes and their main subpopulations in basal conditions (medium) and after PMA stimulation. ↑ = Increased in our study; ↓ = decreased in our study; ___ = No variation.

	CD8 Lymphocytes		CD8 Naïve		CD8 Effector		CD8 Effector Memory		CD8 Central Memory	
	*Medium*	*PMA*	*Medium*	*PMA*	*Medium*	*PMA*	*Medium*	*PMA*	*Medium*	*PMA*
**IFN-γ**	**↑** in SCI-LCP when compared to HC	__________	**↑** in SCI-LCP when compared to HC	__________	__________	**↑** in SCI-ECP when compared to SCI-SP	__________	**↓** in SCI-LCP when compared to SCI-ECP	__________	__________
**IL-10**	**↑** in SCI-LCP when compared to HC and SCI-SP	**↑** in SCI-LCP when compared to HC	**↑** in SCI-LCP when compared to HC	**↑** in SCI-LCP when compared to HC	**↑** in SCI-LCP when compared to HC and SCI-SP	**↑** in SCI-LCP and SCI-ECP when compared to HC	**↑** in SCI-LCP when compared to HC	**↑** in SCI-LCP and SCI-SP when compared to HC	**↑** in SCI-LCP when compared to HC	**↑** in SCI-LCP, SCI-ECP, and SCI-SP when compared to HC
**IL-17**	__________	**↑** in SCI-LCP when compared to HC	**↑** in SCI-LCP when compared to HC	**↑** in SCI-LCP when compared to HC	__________	__________	__________	__________	__________	**↑** in SCI-LCP and SCI-ECP when compared to HC
**IL-9**	**↑** in SCI-LCP when compared to HC	**↑** in SCI-LCP, SCI-ECP, and SCI-SP when compared to HC	**↑** in SCI-LCP and SCI-ECP when compared to HC	**↑** in SCI-LCP, SCI-ECP, and SCI-SP when compared to HC	**↑** in SCI-LCP and SCI-SP when compared to HC	**↑** in SCI-LCP, SCI-ECP, and SCI-SP when compared to HC	**↑** in SCI-LCP when compared to HC and SCI-ECP	**↑** in SCI-LCP and SCI-SP when compared to HC	**↑** in SCI-LCP when compared to HC	**↑** in SCI-LCP and SCI-ECP when compared to HC
**TNF-α**	__________	**↑** in SCI-SP when compared to HC	**↑** in SCI-LCP and SCI-ECP when compared to HC	**↑** in SCI-LCP when compared to HC	**↑** in SCI-ECP when compared to SCI-SP and SCI-LCP	__________	__________	__________	**↑** SCI-ECP and SCI-SP when compared to HC and SCI-LCP (also **↑** relative to HC)	**↑** in SCI-SP when compared to HC and with SCI-LCP
**IL-2**	__________	__________	__________	__________	**↑** in SCI-LCP when compared to HC	__________	**↑** in SCI-LCP when compared to HC	__________	**↑** in SCI-LCP when compared to HC	__________

**Figure A1:**
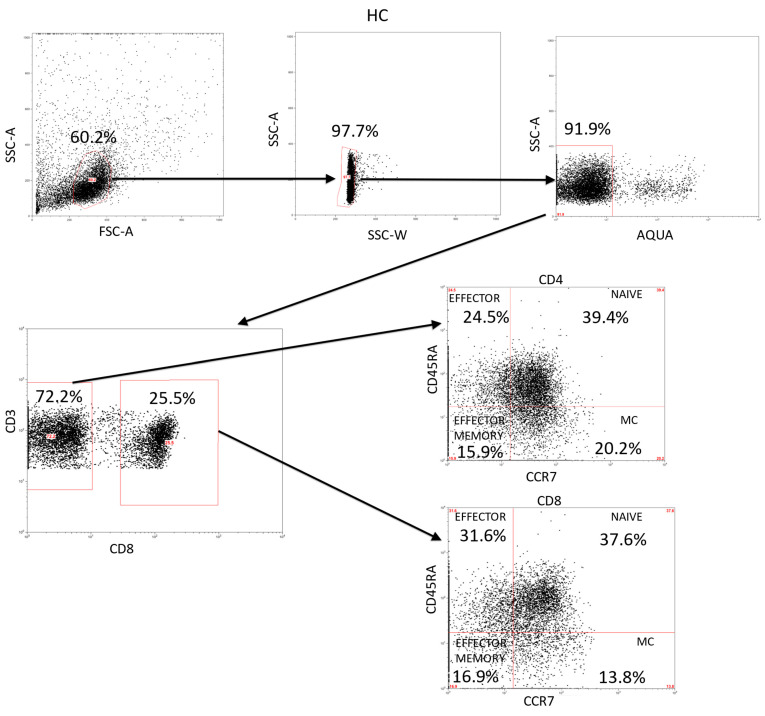
Analysis of CD3+ lymphocytes and their activation/differentiation stages in HC. This is a representation of the gating strategy used for the study of the different subsets in the CD3+ lymphocytes population.

**Figure A2:**
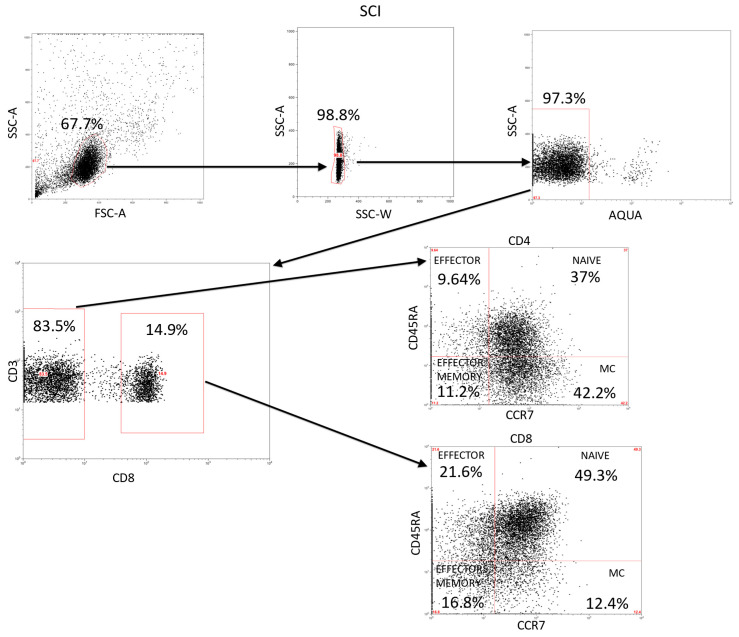
Analysis of CD3+ lymphocytes and their activation/differentiation stages in SCI. This is a representation of the global gating strategy we used for the analysis of the CD4+ and CD8+ populations and the different subsets.

**Figure A3:**
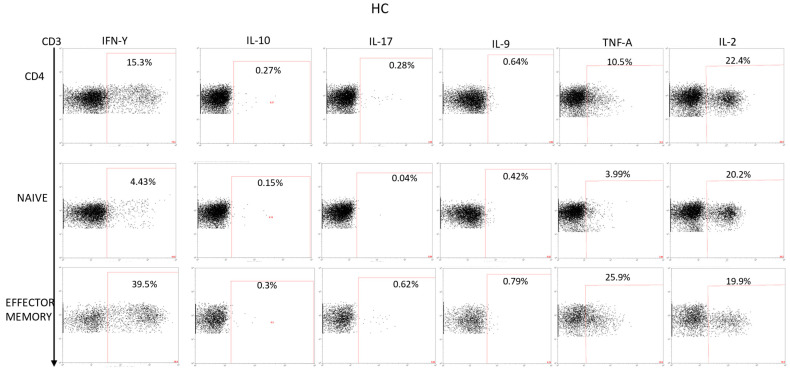
Production of cytokines studied in CD4+, T naive, and T effectors memory populations in HC PBMCs stimulated with PMA + ionomycin. Dot plots represent an example of the gating strategy used to analyze the cytokines production in all the populations in the study.

**Figure A4:**
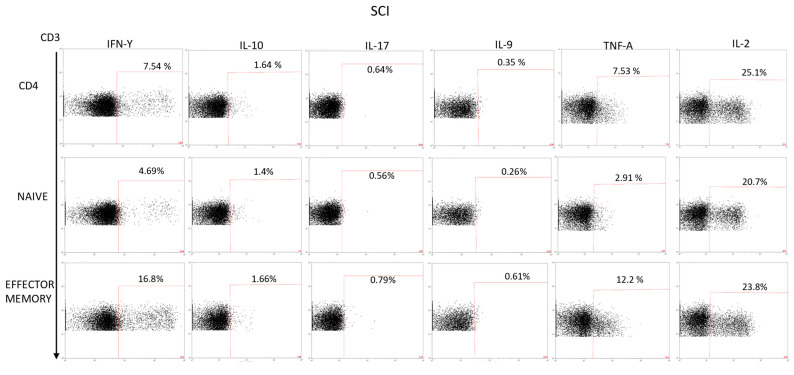
Analysis of the cytokines production of PBMCs stimulated with PMA + ionomycin in the CD4+, naive, and effector memory population. This is an example of the gating strategy we used for all the populations studied in the article.

## Data Availability

The data used to support the findings of the present study are available from the corresponding author upon request.
